# Robust radiosensitization of hemoglobin-curcumin nanoparticles suppresses hypoxic hepatocellular carcinoma

**DOI:** 10.1186/s12951-022-01316-w

**Published:** 2022-03-05

**Authors:** Ruoling Gao, Yuan Gu, Ying Yang, Yuping He, Wenpeng Huang, Ting Sun, Zaixiang Tang, Yong Wang, Wei Yang

**Affiliations:** 1 State Key Laboratory of Radiation Medicine and Protection, School of Radiation Medicine and Protection, Collaborative Innovation Center of Radiation Medicine of Jiangsu Higher Education Institutions, Soochow University, Suzhou, 215123 Jiangsu China; 2 The Fourth Affiliated Hospital, Zhejiang University School of Medicine, Yiwu, 322000 Zhejiang China; 3 Neurosurgery and Brain and Nerve Research Laboratory, The First Affiliated Hospital of Soochow University, Suzhou, 215006 Jiangsu China; 4 Department of Biostatistics, School of Public Health, Medical College of Soochow University, Suzhou, 215123 China

**Keywords:** Hemoglobin, Curcumin, Nanoparticles, Radiotherapy, Hypoxia, Hepatoma

## Abstract

**Background:**

Radioresistance inducing by hypoxic microenvironment of hepatocellular carcinoma is a major obstacle to clinical radiotherapy. Advanced nanomedicine provides an alternative to alleviate the hypoxia extent of solid tumor, even to achieve effective synergistic treatment when combined with chemotherapy or radiotherapy.

**Results:**

Herein, we developed a self-assembled nanoparticle based on hemoglobin and curcumin for photoacoustic imaging and radiotherapy of hypoxic hepatocellular carcinoma. The fabricated nanoparticles inhibited hepatoma migration and vascular mimics, and enhanced the radiosensitivity of hypoxic hepatoma cells in vitro via repressing cell proliferation and DNA damage repair, as well as inducing apoptosis. Benefit from oxygen-carrying hemoglobin combined with polyphenolic curcumin, the nanoparticles also effectively enhanced the photoacoustic contrast and the efficacy of radiotherapy for hepatocellular carcinoma in vivo.

**Conclusions:**

Together, the current study offered a radiosensitization platform for optimizing the efficacy of nanomedicines on hypoxic radioresistant tumor.

**Graphical Abstract:**

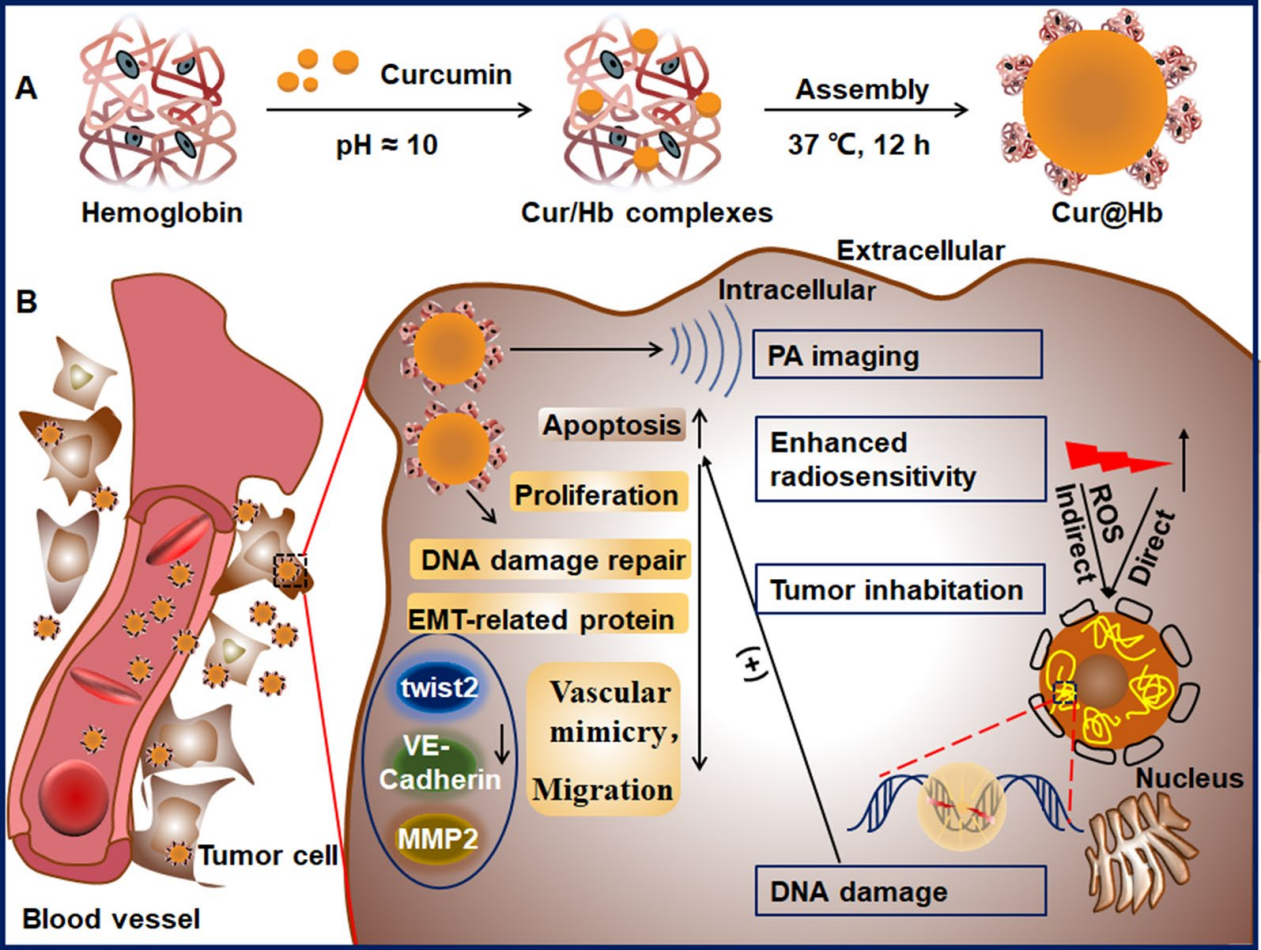

**Supplementary Information:**

The online version contains supplementary material available at 10.1186/s12951-022-01316-w.

## Introduction

According to 2018 statistics worldwide, liver cancer is the sixth common cancer and the fifth common cause of cancer-related death [1]. 80–90% of primary liver cancer is hepatocellular carcinoma (HCC) [2]. As one of the most common clinical treatment modalities, radiotherapy (RT) kills cancer cells via direct and indirect effect [3]. Radiation can directly disrupt DNA structure resulting in cell necrosis or apoptosis [4], while indirect effect of radiation is mediated by reactive oxygen species (ROS) [5], which derive from the interaction of radiation with water and oxygen in the tumor microenvironment. However, most cancer patients undergoing RT suffer from hypoxia inducible factor-1 (HIF-1)-dependent radioresistance. HIF-1 plays a pivotal role in adaptive responses to hypoxia by modulating various cellular functions, including proliferation, apoptosis, angiogenesis, pH balance, and anaerobic glycolysis, which in turn induces tumor radioresistance [6]. In this case, high dose radiation is employed to enhance the efficacy of RT, but it will damage normal tissues. Therefore, radiosensitizers, which can enhance the sensitivity of cancer cells to radiation, are introduced to overcome these obstacles.

It is well-known oxygen acts as a radiosensitizer via radiation-induced damage fixation based on its high electron affinity [7]. To increase the oxygen content in tumor, one method is hyperbaric oxygen treatment [8]. However, it is difficult and cumbersome in practice, and have side effects under certain conditions [9]. Another method is utilizing oxygen carriers, including hemoglobin [10] and perfluorocarbons [11]. The effect of perfluorocarbons is limited when used alone. It shows benefits only when combined use with carbogen breathing. So far, it appears that modulation of the hemoglobin level during radiotherapy is prospective, but rarely reported.

Owing to the very short lifetime and diffuse distance of ROS, ROS produced in the cytoplasm cannot act on the DNA in nuclei [12]. Thus, radiosensitizers delivered into nuclear can generate abundant ROS to attack DNA molecule nearby directly, which might enhance the efficacy of RT. Curcumin, a classic polyphenol molecule, is a robust ROS inducing agent, and also an inhibitor of tumor initiation, proliferation, angiogenesis, and metastasis [13]. It has been reported that curcumin had radiosensitizing effect on cancer cells via inactivating signal transduction pathways, such as signal transducers and activators of transcription (STAT) and nuclear factor-kB (NF-kB) signaling pathways [13]. Moreover, in order to further improve the effectiveness of curcumin administration, novel technologies for curcumin encapsulation is making new progress [14] .

In this study, we fabricated the Cur@Hb nanoparticles in order to combine the radiosensitizing effect of hemoglobin and curcumin on hypoxic hepatocellular carcinoma. Our data showed that Cur@Hb nanoparticles inhibited tumor cell migration and vascular mimics via inhibiting the expression of EMT-related proteins Twist1, VE-Cadherin and MMP2, and enhanced the radiosensitivity of hypoxic hepatocellular carcinoma through inhibiting cell proliferation, promoting apoptosis and increasing DNA damage. Our data provided a feasible idea for radiosensitizing of hypoxic hepatocellular carcinoma.

## Results

### Characterization of Cur@Hb nanoparticles

**Fig. 1:**
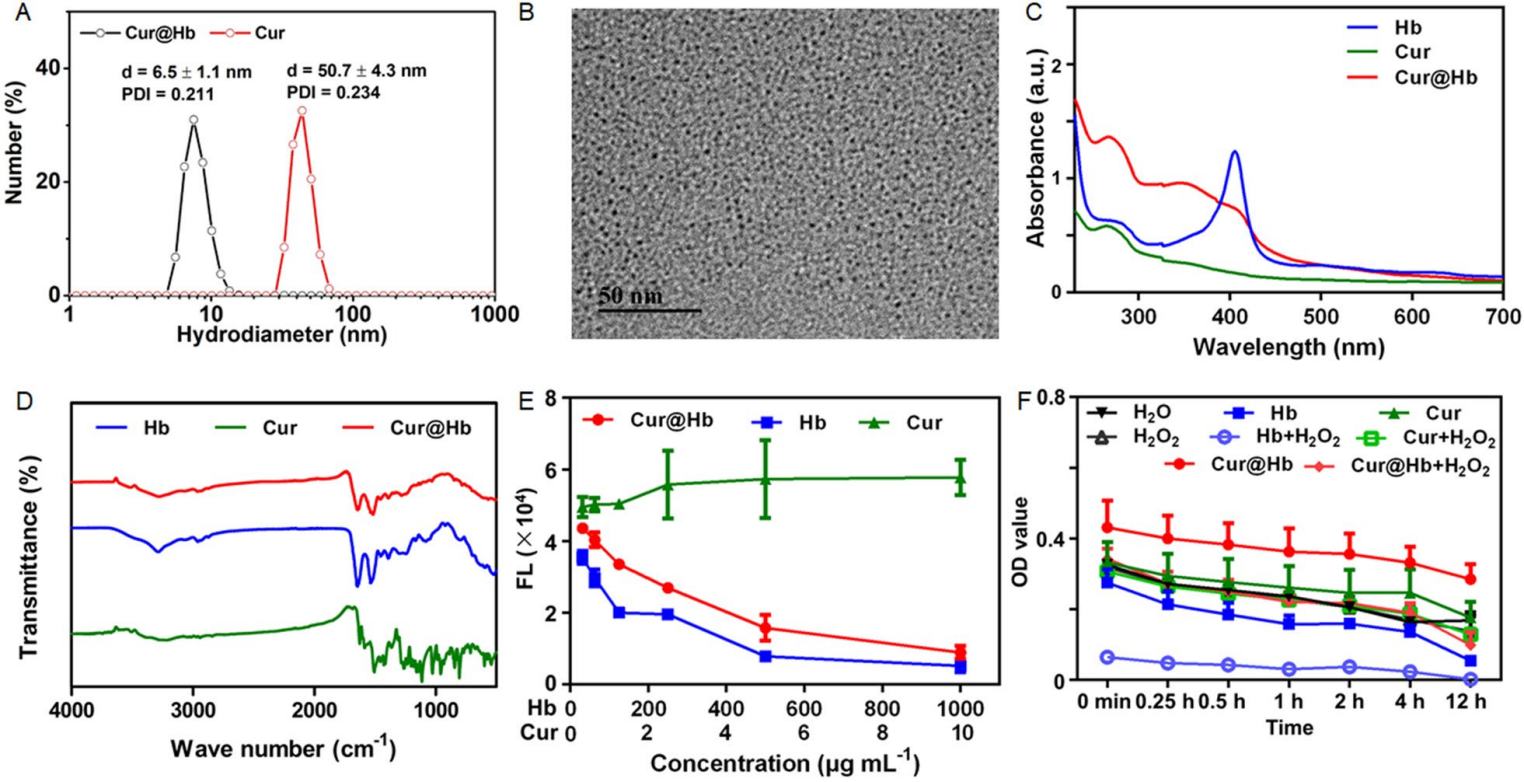
Characterization of Cur@Hb nanoparticles. **A** The hydrated particle size of Cur@Hb nanoparticles. **B** The TEM image of Cur@Hb nanoparticles. **C** The UV spectrum of Cur@Hb nanoparticles. **D** The infrared spectrum of Cur@Hb nanoparticles. **E** ROS production of Hb, Cur and Cur@Hb. **F** ROS production efficiency of Cur@Hb;

The hydrated particle size of Cur@Hb nanoparticles synthesized and used in this study is between 3 and 7 nm (Fig. 1A, B). It had good dispersion and dispersibility in aqueous solution, PBS, PBS containing 10% FBS and DMEM containing 10% FBS (Additional file 1: Fig. S1). Hb had an absorption peak at 406 nm, Cur had an absorption peak at 265 nm, and Cur@Hb had absorption peaks at 265 nm and 406 nm (Fig. 1C). FTIR spectroscopy also confirmed the presence of Hb on the surface of nanoparticles in the fingerprint region of ∼ 1700 nm (Fig. 1D). Also, the loading efficiency of Cur was about 18.5%, which was calculated by establishing the standard curve of curcumin (the calculation method is described in “Materials and methods”). The result was not unsatisfactory considering the method used was protein biomimetic mineralization without organic system. Then, a simulation system in vitro was established, in which PBS at pH6.8 was set up to mimic the tumor microenvironment for drug release. It showed above 60% of curcumin was released from Cur@Hb at 72 h (Additional file 1: Fig. S2).

Cur@Hb catalyzed the generation of ROS in a concentration-dependent manner, that was the higher the concentration of Cur@Hb, the more ROS was produced, so was Hb, whose ROS production was more than Cur@Hb. As the Cur concentration increased, the amount of ROS decreased. After the concentration reached 5 μg/mL, ROS production tended to stabilize (Fig. 1E). In order to detect the ROS production efficiency of Cur@Hb, 250 μg/mL Cur@Hb, 250 μg/mL Hb, 2.5 μg/mL Cur were selected and 10 M H_2_O_2_ was added, then immediately subjected to 4 Gy X-ray irradiation. It was found that after adding H_2_O_2_ to Cur@Hb and Hb groups, ROS production increased significantly, but if H_2_O_2_ was added to the Cur group, ROS production did not change significantly (Fig. 1F). Therefore, the results showed that Cur@Hb and Hb had catalase-like activity.

In order to verify the feasibility of Cur@Hb nanoparticles in photo-acoustic imaging in vivo, we quantitatively analyzed the photo-acoustic images (Additional file 1: Fig. S3). There was a clear linear relationship between the concentration of nanoparticles and the photoacoustic signal, that was, with the concentration of nanoparticles increased, the photoacoustic signal increased significantly. The results suggested that Cur@Hb nanoparticles have application prospects in photoacoustic imaging.

### Effect of Cur@Hb on cell viability and cell uptake

**Fig. 2:**
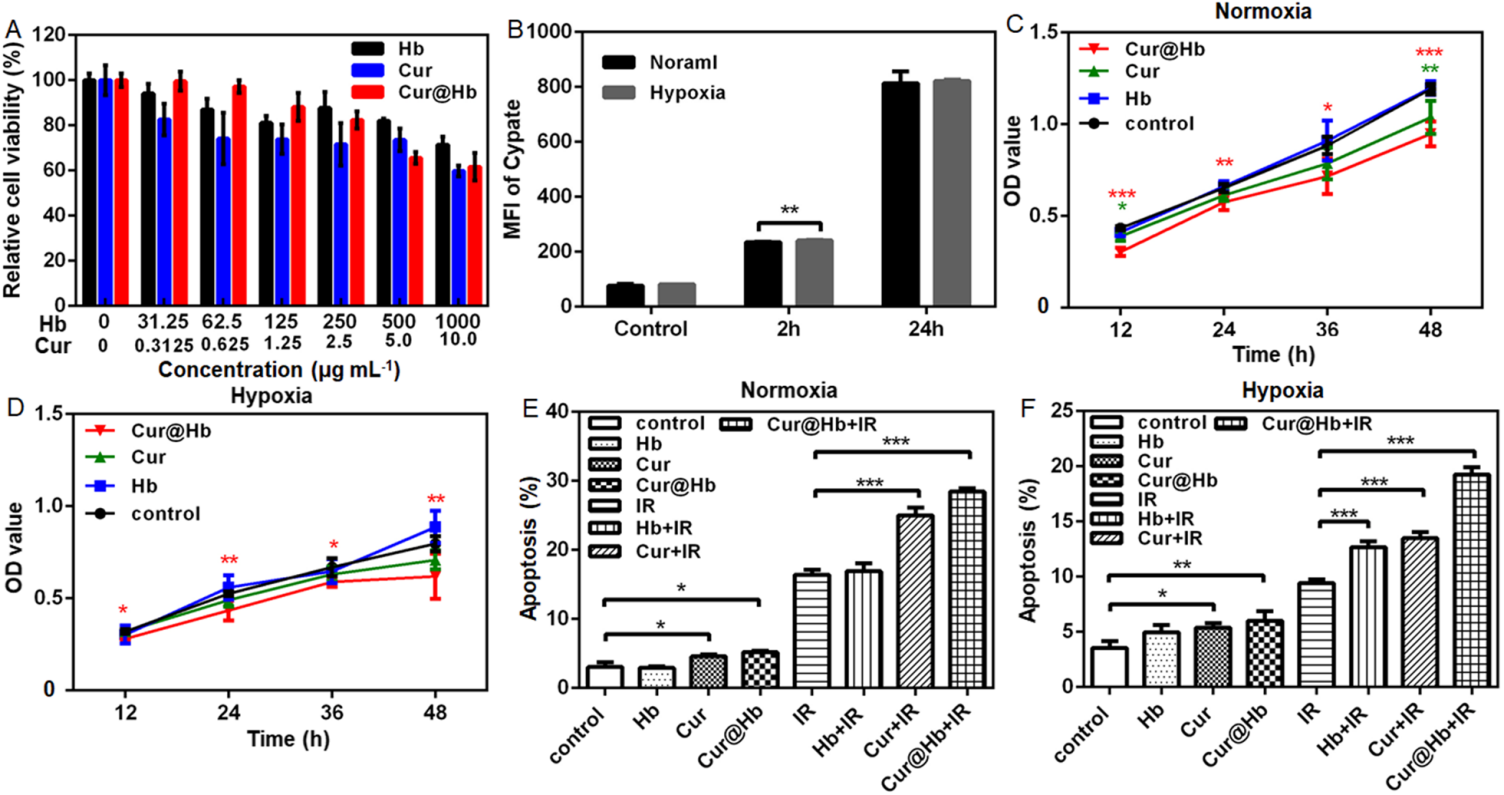
Effects of Cur@Hb nanoparticles on SMMC7721 cell proliferation, cell cycle, and apoptosis. **A** Cytotoxic effects of Hb, Cur and Cur@Hb on SMMC7721 cells. **B** Quantitative analysis of fluorescence intensity. **C**, **D** Effects of Hb, Cur and Cur@Hb nanoparticles on cell proliferation under normoxia or hypoxia. **E**, **F** Effects of Hb, Cur and Cur@Hb nanoparticles on cell apoptosis under normoxia or hypoxic condition, with or without 4 Gy irradiation. Data are representative of three independent experiments and expressed as mean ± SD (n = 3, technical replicates), one-way ANOVA followed by Bonferroni post-test, *p < 0.05, **p < 0.01, and ***p < 0.001

In order to determine an appropriate concentration for subsequent experiments, we tested the material's toxicity to SMMC7721 cells by CCK-8 experiment. Cells were incubated with different concentrations of Hb, Cur, and Cur@Hb nanoparticles for 24 h, then subjected to CCK-8 assay. All nanoparticles induced no obvious toxicity to SMMC7721 cells even at 100 μg/mL Hb, Cur@Hb and 2.5 μg/mL Cur (Fig. 2A). The results indicated that Cur@Hb nanoparticles reduced the curcumin toxicity and increased the water solubility of curcumin.

To determine whether there was difference in the uptake of the nanoparticles by SMMC 7721 cultured under normoxic and hypoxic conditions, we used qualitative laser confocal detection and flow cytometry analysis. Additional file 1: Fig. S4A and Fig. 2B showed the addition of Cypate-labeled Cur@Hb after 2 h and 24 h culture in normoxic or hypoxic environment, respectively. The lysosomes were stained, and Cur@Hb was taken up by the lysosomes. After 24 h, there was no significant difference between normoxic and hypoxia intakes. Through the quantitative analysis of fluorescence intensity, we found that after adding Cur@Hb for 2 h, the difference in fluorescence intensity between normoxic and hypoxic cultures was statistically significant, but it was not significant for 24 h (Fig. 2B). Additional file 1: Fig. S4B, C showed fluorescence peaks measured by flow cytometry in normoxic and hypoxic environments, respectively. What we wanted to testify was whether it was the own nature of the Cur@Hb nanoparticles that caused the different effects in the normoxic and hypoxic environment, rather than the difference in the amount of endocytosis between normoxic and hypoxic environment, therefore, 24 h was selected as the incubation time in subsequent experiments according to these data.

### Cur@Hb inhibited cell proliferation and promoted apoptosis

We used SMMC7721 cells in logarithmic growth phase, and added 250 μg/mL Hb 100 μL, 2.5 μg/mL Cur 100 μL, 250 μg/mL Cur@Hb 100 μL, and 100 μL basal medium to each well. After culturing for 12 h, 24 h, 36 h, and 48 h under normoxic and hypoxic conditions, the effects of Hb, Cur, Cur@Hb on normoxic or hypoxic human hepatoma cell proliferation were investigated by CCK-8 assay (results were showed by OD value). The results showed that Cur@Hb nanoparticles decreased the proliferation of normoxic or hypoxic SMMC7721 cells after 12, 24, 36 and 48 h incubation (*P* < 0.05 or *P* < 0.01). In addition, Cur decreased proliferation of normoxic SMMC7721 cells after 12 and 48 h incubation (Fig. 2C, D). These results indicated that Cur@Hb nanoparticles can suppress normoxic or hypoxic human hepatoma cell proliferation.

The above results confirmed that Cur@Hb could inhibit the proliferation of SMMC7721 cells under normoxic and hypoxic conditions. To investigate whether Cur@Hb inhibited cell proliferation through affecting the cell cycle progression or not, we detected the cell cycle by flow cytometry after normoxic or hypoxic culture for 24 h. Compared with the control group, Hb + IR, Cur + IR and Cur@Hb + IR group caused G2/M cell cycle arrest (*P* < 0.05). In particular, Cur@Hb + IR group caused more obvious G2/M cell cycle arrest (*P* < 0.001). The results showed that these materials alone did not affect the progress of the cell cycle (Additional file 1 Fig. S5A, B), and X-ray irradiation was the main cause of G2/M phase arrest.

Since the treatment of individual materials had no effect on the cell cycle progression, we wonder whether Cur@Hb affected cell proliferation by increasing cell apoptosis. Therefore, we used flow cytometry to examine the effect of these three materials on the apoptosis of SMMC7721 cells under normoxic and hypoxic culture. Compared with the control group, the material alone group increased apoptosis except Hb. Combined with X-ray irradiation, the Cur group and Cur@Hb group significantly increased the apoptosis rate (*P* < 0.001). More importantly, the apoptosis rate of the Cur@Hb group was significantly higher than that of the Cur group and the Hb group under both culture conditions (Fig. 2E, F; Additional file 1: Fig. S6). The results indicated that Cur@Hb could promote human hepatoma cell apoptosis when administered alone or combined with X-ray irradiation.

### Cur@Hb inhibited the migration and vascular mimicry of normoxic and hypoxic hepatoma cells

**Fig. 3:**
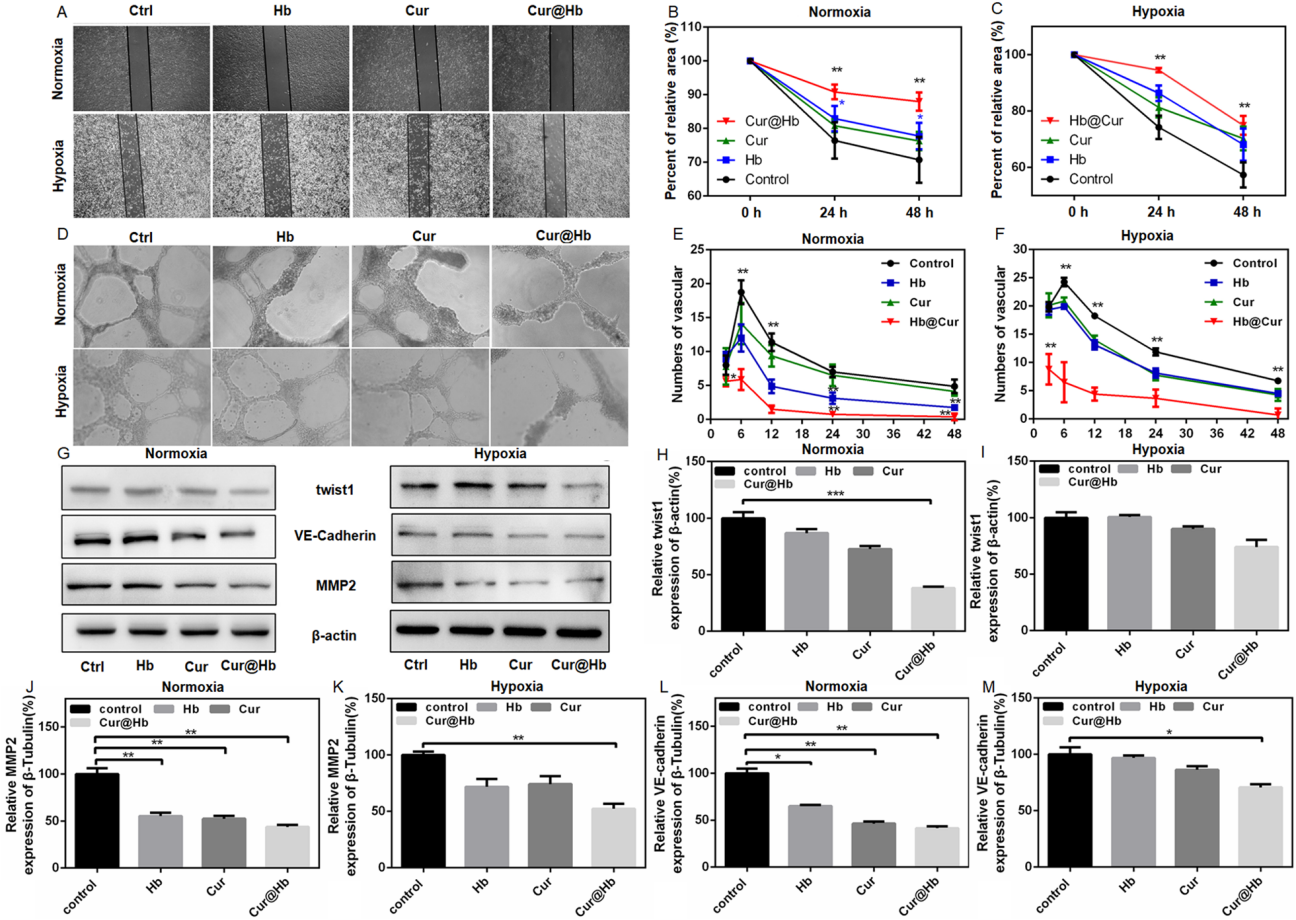
Effects of Cur@Hb nanoparticles on SMMC7721 cell migration and vascular mimicr. **A**–**C** Effect of three materials on cell migration under normoxia or hypoxia. **D**–**F** Effect of three materials on vascular mimicry under normoxia or hypoxia. **G**–**M** Effects of Hb, Cur and Cur@Hb nanoparticles on the expression of twist1 (H and I), MMP2 (**J**, **K**) and VE-cadherin (**L**, **M**) under normoxia or hypoxia

Migration is one of the important malignant behaviors of cancer cells. In order to explore whether Cur@Hb could affect the migration ability of hepatoma cells, SMMC7721 cells were incubated under normoxia or hypoxia (1% O_2_) for 0, 24, and 48 h, then photographed to calculate the scratch area. The results showed that Cur@Hb could significantly inhibit the migration of tumor cells at 24 h and 48 h under normoxia or hypoxia culture (Fig. 3A–C; Additional file 1: Fig. S7A, B).

Vasculogenic mimicry (VM) is a network of neovascularization formed by highly invasive tumor cells instead of endothelial cells [15]. We cultured SMMC7721 cells under normoxia or hypoxia (1% O_2_) condition to observe changes in the number of vascular lumen-like structures at various time points. The experimental results showed that Cur@Hb inhibited the formation of lumen-like structures as early as 3 h after culture (Fig. 3D–F; Additional file 1: Fig. S7C, D). In addition, we detected the expression of EMT-related proteins by western blot. Cur@Hb inhibited the expression of twist1, VE-Cadherin, and MMP-2 in SMMC7721 cells under normoxia or hypoxia condition (Fig. 3G–M). These results suggested that Cur@Hb could inhibit VM by reducing the expression of EMT-related proteins.

### Cur@Hb enhanced radiosensitivity of hypoxic hepatoma cells

**Fig. 4:**
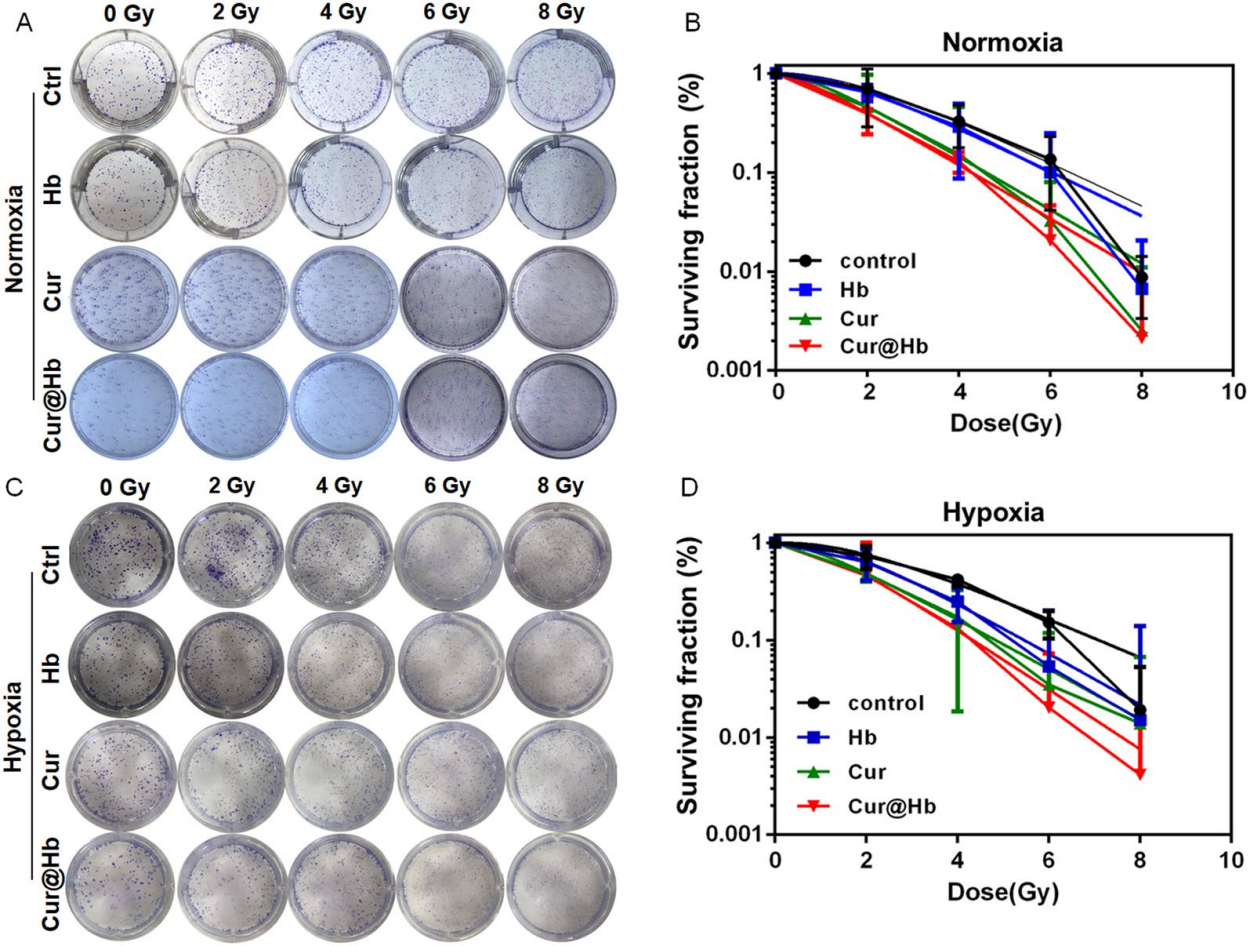
Effects of Cur@Hb nanoparticles on SMMC7721 cell radiosensitivity. **A**, **B** Cur@Hb nanoparticles enhanced the radiosensitivity of normoxic SMMC7721 cells. **C**, **D** Cur@Hb nanoparticles enhanced the radiosensitivity of hypoxic SMMC7721 cells

Radiosensitivity refers to how quickly different bodies, tissues, organs, cells respond to ionizing radiation damage and death under the same irradiation conditions. We used clone survival experiments to verify whether the nanoparticles had radiosensitizing effect on SMMC7721 cells. The results of clonogenic assay showed that normoxic SMMC7721 cells treated with Hb, Cur and Cur@Hb (D_0_ = 1.903, 1.594, 1.586 Gy) were more sensitive to X-ray irradiation than control cells (D_0_ = 1.962 Gy), and the radiosensitizing ratio were 1.030, 1.231 and 1.236, respectively (Fig. 4A, B). Under hypoxic conditions, SMMC7721 cells treated with Hb, Cur and Cur@Hb (D_0_ = 1.860, 1.874, 1.636 Gy) were more sensitive to X-ray irradiation than control cells (D_0_ = 2.470 Gy), and the radiosensitization ratio were 1.328, 1.318 and 1.510, respectively (Fig. 4C, D). The oxygen enhancement ratio (OER) was calculated as the ratio of D_0_ (hypoxia) to D_0_ (normoxia). The OER value of control cells and cells treated with Cur@Hb was 1.236 and 1.510, respectively. These results suggested that curcumin-hemoglobin nanoparticles improve the radiosensitivity of SMMC7721 cells not only under normoxic conditions, but also under hypoxic conditions.

### Cur@Hb inhibited DNA damage repair of normoxic and hypoxic hepatoma cells

**Fig. 5:**
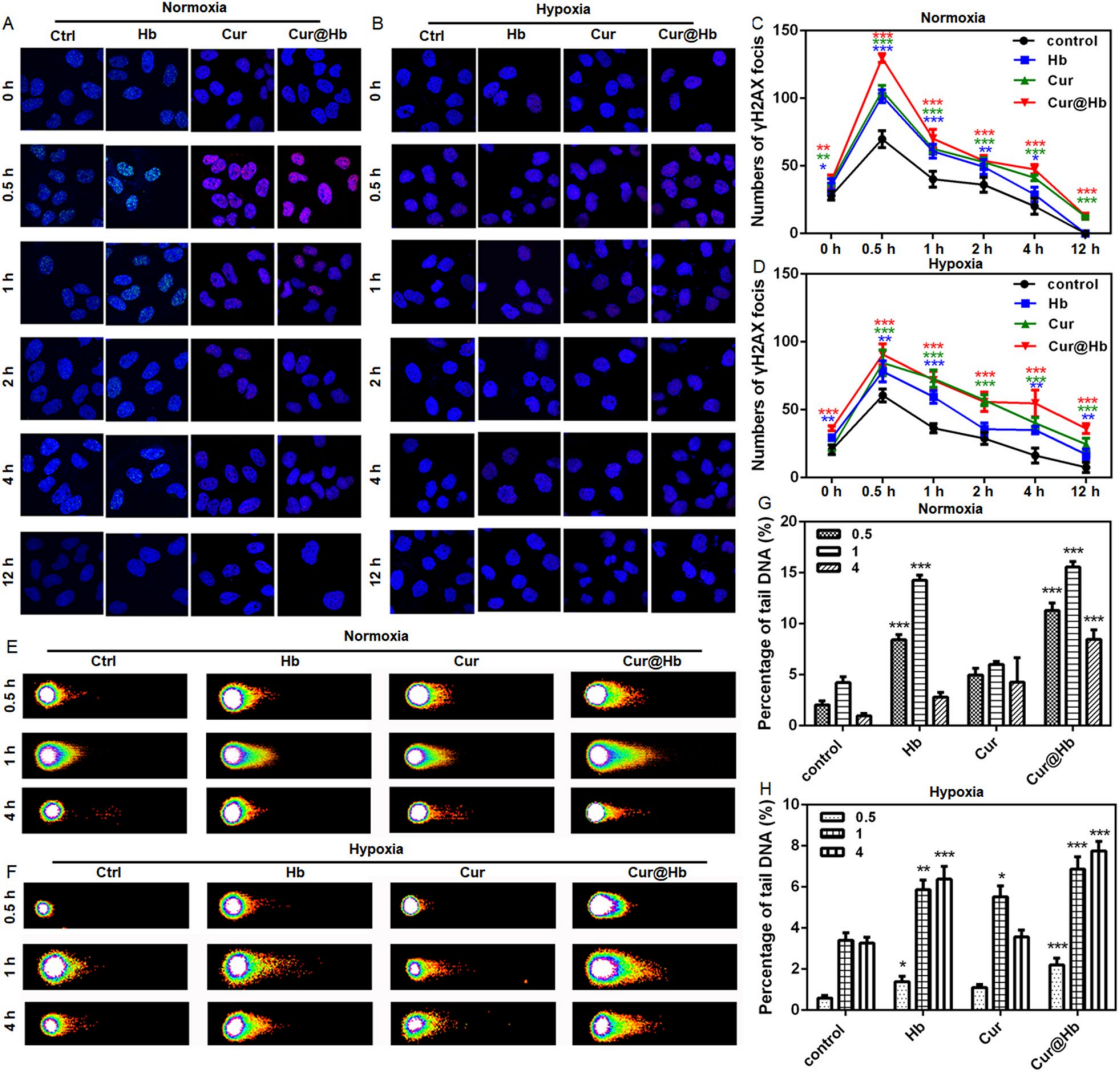
Effects of Cur@Hb nanoparticles on the DNA damage of SMMC7721 cells induced by 4 Gy X-ray. A-D: Cur@Hb nanoparticles aggravated DNA damage during normoxia (**A**, **C**) and hypoxia (**B**, **D**); **E**, **F** Pictures of single cell gel electrophoresis. **G**, **H** Histogram of quantitative analysis of comet assay under normoxia (**E**) or hypoxia (**H**). Data are representative of three independent experiments and expressed as mean ± SD, one-way ANOVA followed by Bonferroni post-test, *p < 0.05, **p < 0.01, and ***p < 0.001

DNA is an important target molecule for ionizing radiation. In order to measure the DNA damage induced by 4 Gy X-ray alone or in combination with nanomaterials, we used immunofluorescence assay to detect the expression of γH2AX. Cur@Hb nanoparticles not only increased the peak value of γH2AX foci produced by X-ray, but also extended the duration of damage (Fig. 5A–D; Additional file 1: Fig. S8). Comparing the experimental results of normoxia and hypoxia, it was found that the number of γH2AX foci under hypoxic condition was lower than that under normoxic condition at points less than one hour (Fig. 5C, D), which was in line with hypoxia leading to increased radiation resistance of tumor cells [16]. But over time, the Cur@Hb nanoparticles induced hypoxic cells to produce more γH2AX foci than normoxic cells, the Cur@Hb nanoparticles induced the hypoxic cells to produce about twice as much γH2AX foci as the normoxic cells at 12 h. Further, the results of single cell gel electrophoresis were basically in accord with the results of γH2AX immunofluorescence staining (Fig. 5E–H). Due to the different experimental methods, in the single-cell gel electrophoresis experiment, the DNA damage was most severe at 1 h after exposure, as shown in Fig. 4E–G. Although different materials had different effects on the DNA content of the tail of the comet, they all increased the DNA content of the tail, particularly Cur@Hb. These results indicated that Cur@Hb could increase DNA damage of hepatoma cells induced by X-ray, thereby enhancing the killing effect of X-ray.

### Cur@Hb increased the production of ROS in hepatoma cells and promoted the polarization of macrophages from M2 to M1.

We used the fluorescent probe DCFH-DA to detect the ROS level in living cells. The results showed that ROS levels of Hb group or Cur group were lower than that of control group (*P* = 0.02 in Hb group and *P* < 0.001 in Cur group), but ROS level of Cur@Hb group was not statistically different from that of control group at 24 h after adding materials. In addition, Cur@Hb increased ROS production at 6 h, 12 h, 48 h and 72 h post-irradiation, while Cur group increased ROS production only at 48 h after irradiation, compared with control group (Additional file 1: Fig. S9A). The above results suggested that Cur@Hb combined with X-ray irradiation could increase ROS production in hepatoma cells to achieve a stronger tumor killing effect.

Macrophages are involved in tissue homeostasis, defense mechanisms and wound healing [17]. They also play a role in various diseases, such as autoimmune diseases, atherosclerosis and tumorigenesis [18, 19]. Macrophages polarized to M1 type can inhibit the progression and metastasis of cancer, while macrophages polarized to M2 type can promote cancer development [20]. In the experiment, we used IL-4 pretreatment to polarize RAW264.7 macrophages to M2 type, and then added different materials, with LPS as a positive control. The experimental results showed that Cur@Hb could significantly promote the polarization of macrophages from M2 to M1 (M1/M2 = 0.415 ± 0.01997; LPS group M1/M2 = 0.538 ± 0.01119, LPS is a positive control) (Additional file 1: Fig. S9B). ELISA kits were used to detect the concentration of TNF-α in cell culture supernatant under different treatment conditions. The results showed that Cur@Hb nanoparticles increased the concentration of TNF-α to about 700 pg/mL, almost 1.5 times more than that of control group (Additional file 1: Fig. S10). The increased TNF-α helped macrophages to exert better tumor-killing effects.

### Cur@Hb inhibited the growth of transplanted tumors in nude mice

**Fig. 6:**
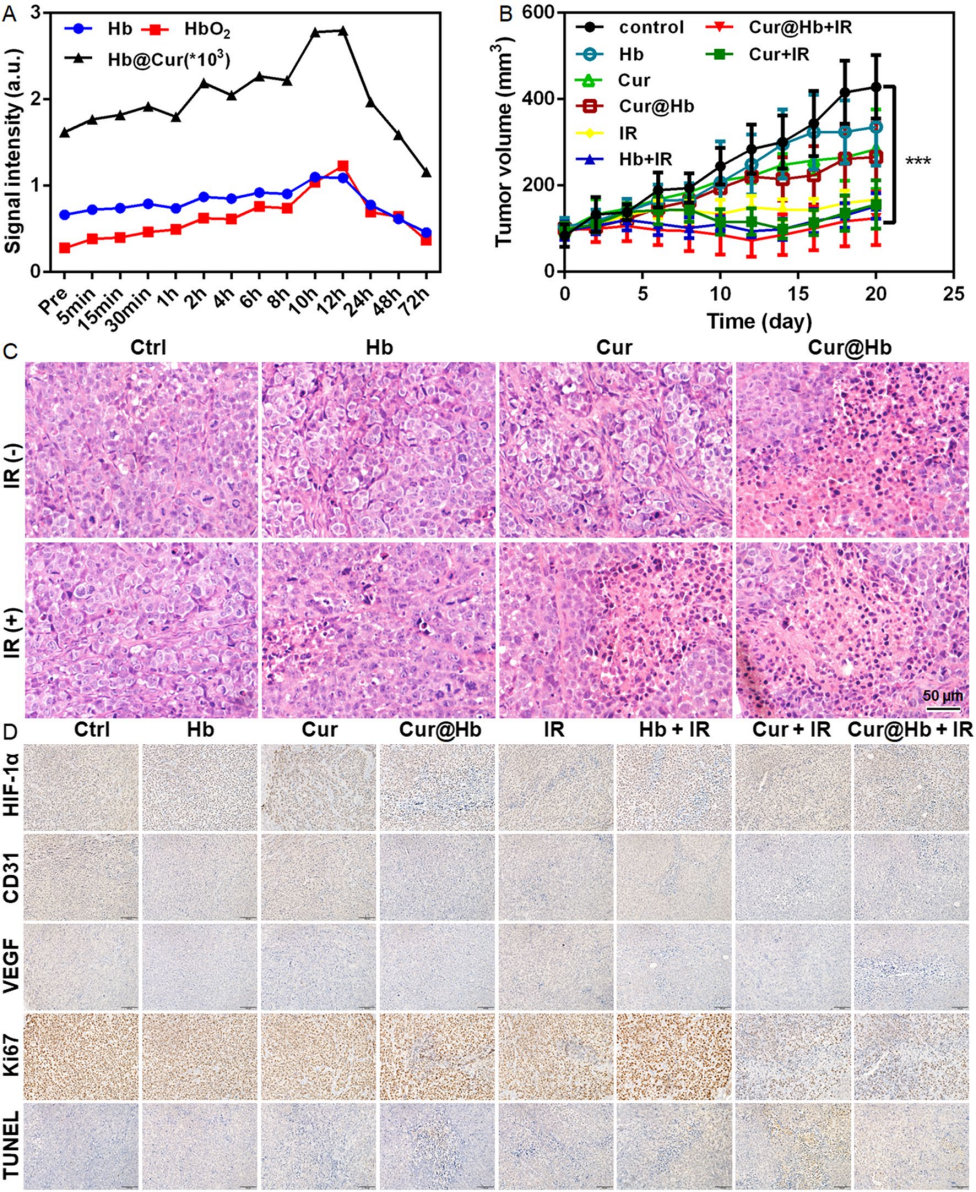
In vivo experiments of Cur@Hb nanoparticles. **A** Quantitative analysis of the content of intratumor hemoglobin, oxyhemoglobin, and Cur@Hb nanoparticles at different time points. **B** Tumor volume of nude mice. **C** Pathological analysis of tumor cells invasion. **D** Representative images for HIF-1α, CD31, VEGF, Ki67 and TUNEL staining of tumor sections. n = 3, one-way ANOVA followed by Bonferroni post-test, ***p < 0.001

In order to determine the time when Cur@Hb reached the maximum enrichment in the tumor, we performed quantitative analysis based on the photo-acoustic image (Fig. 6A; Additional file 1: Fig. S11), the signal of the material in transplanted tumors increased with time, peaked at 12 h, then decreased. In addition, the change trend of hemoglobin content or oxyhemoglobin content in tumors was consistent with that of Cur@Hb nanoparticles. The hemoglobin content increased continuously with time and reached a peak at 12 h, and then decreased with time. These results suggested that the intratumoral material content and oxygenated hemoglobin content reached the peak at 12 h after tail vein injection of nanoparticles. Therefore, radiation therapy can be conducted at this time point, which is not only conducive to fully exerting the radiosensitization effect of Cur@Hb, but also can effectively improve the tumor oxygenation status, and these two aspects may synergistically enhance the radiosensitivity of hypoxic hepatoma.

Through the measurement of the transplanted tumor volume of nude mice, we found that the tumor volume of nude mice in IR group and Cur + IR group was smaller than that in control group (P < 0.05) on days 6–8. On days 10–20, the tumor volume of nude mice in IR group, Hb + IR group, Cur + IR group and Cur@Hb + IR group was significantly smaller than that of control group (P < 0.01 or 0.001). On day 20, the tumor inhibition rates of Hb group, Cur group, Cur@Hb group, IR group, Hb + IR group, Cur + IR group and Cur@Hb + IR group were 21.61%, 33.62%, 38.02%, 60.79%, 64.41%, 63.55% and 71.02%, respectively (Fig. 6B). To confirm the ablation of tumor cells, one nude mouse was sacrificed per group, and the tumor was collected for H&E staining at the end point of the treatment. Pathological analysis revealed less tumor cells invasion in the Cur@Hb + IR group than in other control groups (Fig. 6C). Besides, the representative images for HIF-1α, CD31, VEGF, Ki67 and TUNEL staining of tumor sections (Fig. 6D) reflected that Cur@Hb nanoparticles could alleviate tumor hypoxia, inhibit angiogenesis and tumor cell proliferation and promote tumor cell apoptosis, thereby enhancing the efficacy of radiotherapy for hepatoma. Although there was no significant difference in tumor growth rate between Cur + IR and Cur@Hb + IR groups, we found that Cur@Hb + IR could better inhibit the infiltration of tumor cells than Cur + IR through the pathologic result (Fig. 6C). Besides, Cur@Hb + IR could better inhibit vascular proliferation, tumor cell proliferation, and induce the increase of tumor cell apoptosis through immunohistochemistry results (Fig. 6D). It is possible that the relatively large dose of radiation did not make a significant difference in tumor volume between the two groups of free Cur and Cur@Hb in the initial period we observed, but we believe that there should be an observable difference between the two groups if the mice had survived longer based on the pathological and immunohistochemical results.

Meanwhile, the Cur@Hb nanoparticles were well-tolerated in animal safety studies, without evidence of interference in the body weight of mice among these groups (*P* > 0.05) (Additional file 1 Fig. S12), and without abnormity in organ histology (Additional file 1: Fig. S13).

## Discussion

We have constructed a novel type of radiosensitizer Cur@Hb nanoparticles. A research team has reported that they developed curcumin-loaded hemoglobin nanoparticles (CCM-Hb-NPs) through self-assembly, which were stable and uniform in size. They experimentally confirmed that this self-assembly process was mainly driven by hydrophobic forces. CCM-Hb-NPs had higher cell absorption efficiency and lower cytotoxicity than free CCM in vitro. Uptake inhibition experiments also showed that their nanoparticles were incorporated through the classical clathrin-mediated endocytosis pathway [21]. Although they developed the hemoglobin curcumin nanocomposite for the first time, they did not further explore the biological effects of the nanoparticles.

Curcumin targets multiple pathways (Wnt/β-catenin, Notch, NF-κB, PI3K/Akt/mTOR, MAPK and Hh pathways) to exert its anti-cancer effects [13]. However, there are few reports on the anti-tumor effects of curcumin on hypoxic tumor cells. Mareike Ströfer et al. showed that curcumin decreased HIF-1α and HIF-2α protein levels and the survival rate of hypoxic Hep3B and MCF-7 cells [22]. Sheeja Aravindan et al. showed that IR combined with Cur generated a stronger killing effect on hypoxic breast cancer cells than IR alone [23]. In this study, we also found curcumin significantly promoted the apoptosis of hypoxic SMMC7721 cells, and the apoptosis rate of the Cur@Hb group was significantly higher than that of the Cur group.

As we all know, cell radiosensitivity of different phases of the cell cycle is quite different. For most cells, the G2/M phase is the most radiosensitive period. It has been reported that curcumin can increase G2/M phase cell proportion in breast cancer [24], pancreatic cancer [25], human gliomas [26], non-small cell lung cancer [27], osteosarcoma [28]. However, in this study, neither curcumin treatment alone nor Cur@Hb nanocomposite could affect the cell cycle progression of SMMC7721 cells, perhaps owing to the low dose curcumin we used, compared with the above studies. Although curcumin and Cur@Hb nanocomposite did not hinder the cell cycle progression, they reduced the proliferation of normoxic and hypoxic SMMC 7721 cells by promoting apoptosis. In addition, curcumin can also inhibit tumor cell migration and invasion (non-small cell lung cancer [27], osteosarcoma [28], glioma [29], colorectal cancer [30], retinoblastoma [31]). Similar results were also obtained in this study.

In adults, under physiological conditions, blood vessels remain stationary and rarely form new branches. In order to support the high proliferation rate of cancer cells, tumors rapidly develop new blood vessel networks. However, compared with normal tissue blood vessels, the tumor blood vessel network is dilated, tortuous and chaotic [32]. Some of these abnormal blood vessels are called angiogenic mimicry (VM). VM describes the ability of aggressive tumor cells to trans-differentiate [33]. Studies have shown that twist family bHLH transcription factor 1 (TWIST1) uses auxiliary proteins to open the nuclear membrane pores and enter the nucleus to regulate the transcription of downstream genes involved in the VM process [34]. Matrix metalloproteinases (MMPs) are a family of zinc-binding endopeptidases and are also involved in angiogenesis [36]. In addition, the hypoxic microenvironment has an inducing effect on the formation of hepatoma VM [35, 36]. Curcumin has the ability to inhibit the growth of implanted melanoma VM channels by regulating angiogenesis factors related to EphA2/PI3K/MMPs signaling pathway [37]. In glioblastoma, curcumin inhibits the formation of VM in human glioma U251 cells in a dose-dependent manner by down-regulating the protein and mRNA expression of EphA2, PI3K, and MMPs [38]. In the present study, both curcumin and Cur@Hb nanocomposites inhibited vascular mimicry and the expressions of VE-cadherin, twist1 and MMP2, suggesting Cur@Hb might be used as an anti-VM drug for hepatoma.

Curcumin can increase the radiosensitivity of renal cell carcinoma [39], non-Hodgkin's lymphoma [40], rhabdomyosarcoma [41], oral squamous carcinoma [42] and colon cancer [43]. In addition to the traditional form of curcumin, curcumin in the form of nanoparticles was also documented to play a radiosensitizing role. New curcumin preparations including nanocrystals, liposomes, polymers and micelles, as well as co-delivery of curcumin and adjuvants are all documented to enhance their bio-availability [44]. Nano-curcumin has better anti-tumor effect due to improved bio-availability. Xu et al. developed a new curcumin-based supramolecular nanofiber (Cur-SNF) by self-assembling short peptides. Compared with free curcumin, Cur-SNFs showed better performance as radiosensitizer, and promoted radiation sensitivity of colorectal cancer cells by inhibiting radiation-induced NF-κB activation [45]. To sum up, curcumin and its nano-preparations can improve the radiosensitivity of various types of tumor cells. Does curcumin also play a role in enhancing radiosensitivity of hypoxic tumor cells? It has been reported that some phytochemicals, including curcumin, radiosensitized hypoxic cancer cells through regulating NF-κB signaling pathway [23]. In this study, we found that curcumin nanoparticles coated with Hb could maintain the efficacy of curcumin to enhance the radiosensitivity of normoxic and hypoxic tumor cells. Ogiwara et al. showed that curcumin inhibited DNA repair signal pathways by suppressing histone acetyltransferase and Rad3-related protein (ATR) kinase, suggesting inhibition of DNA repair might be one of the main mechanisms of its radiosensitizing effect [46].

Tumor hypoxia is related to treatment resistance, tumor progression and poor clinical prognosis [47]. The adaptive response to hypoxia mainly depends on hypoxia-inducing factors (HIFs). HIF-dependent signaling make cancer cells and stromal cells adapt to the hypoxic environment, which is beneficial to promote cancer progression [20]. In order to achieve better therapeutic effects, a variety of nanotechnology based on overcoming hypoxia has been developed, including (i) reducing oxygen consumption by inhibiting cellular respiration; (ii) normalizing tumor blood vessels to promote blood flow in the tumor; (iii) bringing exogenous oxygen into the tumor; (iv) generating oxygen in situ [48]. Metformin has been shown to be a drug that effectively inhibits cellular respiration by directly inhibiting the activity of complex I in the mitochondrial electron transport chain [49, 50]. Gold nanoparticles (AuNPs) help normalize tumor vasculature and increase blood perfusion and relieve hypoxia in melanoma tumors [51]. Hemoglobin (Hb) is encapsulated separately into liposomes (Hb-Lipo) to form a bionic oxygen delivery system, which can effectively relieve tumor hypoxia and significantly enhance the efficacy of chemo-radiotherapy [52]. Manganese dioxide nanomaterials can react with H_2_O_2_ in the tumor microenvironment to generate oxygen, reducing tumor hypoxia [53–55]. In this study, we used photo-acoustic imaging to determine the content of hemoglobin and oxyhemoglobin in transplanted tumors. Cur@Hb used hemoglobin as an oxygen carrier to bring exogenous oxygen into the tumor, thereby alleviating hypoxia in the tumor area and enhancing the killing effect of X-rays on tumor cells. We found that Hb, Cur or Cur@Hb combined with X-rays could inhibit the growth of transplanted tumors in nude mice, but there was no statistical difference in tumor volume among Hb + IR, Cur + IR and Cur@Hb + IR groups, which might be due to too large a single exposure dose or too many exposure times. However, we found that Cur@Hb + IR had the largest tumor inhibition rate, indicating that Cur@Hb combined with X-ray had the best therapeutic effect under the same treatment conditions.

Macrophages are crucial populations in tumor stroma, playing key roles in tumorigenesis, growth, metastasis and resistance to radiotherapy and chemotherapy. Tumor-associated macrophages (TAMs) are heterogeneous and plastic, existing as a spectrum of populations between classically activated (M1) type and alternatively activated macrophages (M2) type based on its functional properties [18, 19]. M1-like macrophages secrete IL-12, TNF-α, reactive oxygen species (ROS) and so on, which are often resident in nomorxic region and associated with favorable prognosis in cancer patients [56, 57]. In contrast, M2-like macrophages are pro-tumoral and immunosuppressive, usually accumulated in hypoxic region [58, 59]. In this study, Cur@Hb could obviously promote the polarization of M2-like macrophages toward M1 direction and effectively improve the tumor immunosuppressive microenvironment, which provided another mechanism for the anti-tumor effect of Cur@Hb.

## Conclusion

The results presented here show that Cur@Hb nanoparticles could inhibit hepatoma migration and vascular mimics, and enhance the radiosensitivity of hypoxic hepatoma cells via repressing cell proliferation and DNA damage repair, as well as inducing apoptosis in vitro. Moreover, benefit from the combination of oxygen-carrying hemoglobin with polyphenolic curcumin, the nanoparticles could effectively enhance the photoacoustic contrast and better inhibit the infiltration of tumor cells in vivo, which laid a theoretical and experimental basis for the application of Cur@Hb nanomaterials as radiosensitizers for hepatoma radiotherapy and might provide a valuable type of nano-radiosensitizer for radiotherapy of solid tumors. It is worthy of further study to optimize the radiosensitization treatment scheme to improve the efficacy of radiotherapy.

## Materials and methods

*Synthesis Cur@Hb nanoparticle:* 2.5 mL 100 mM curcumin solution (pH ≈10) was added into 50 mL of hemoglobin (5 mg/mL) solution, the mixture was stirred at 37 °C for 12 h, and dialyzed to ultrapure water in 100 kDa membrane for 24 h. The production was lyophilized to powder and stored at 4 °C until use.

### Loading efficiency

Firstly, a portion of Cur@Hb solution was freeze-dried into powder and then the powder was weighed. Next, the free curcumin was diluted into different concentrations and the absorption peaks at 265 nm were measured and a standard curve with good linearity between absorption and concentration was established. Then, the powder of Cur@Hb was redissolved into a liquid, and measured the absorption value at 265 nm of the Cur@Hb solution, and put it into the established standard curve to obtain the concentration of curcumin in the Cur@Hb liquid. The mass of curcumin was then obtained from multiplying the volume of Cur@Hb solution by the concentration of the curcumin. The result of loading efficiency was expressed as the ratio of the mass of cuecumin to the mass of Cur@Hb powder.b

### Drug release

In order to examine the drug release in vivo, an in vitro model was established. Firstly, 10 mL PBS at pH 6.8 was added into the 50 mL centrifuge tube in order to simulate the pH of tumor microenvironment. Then, 1 mL Cur@Hb, in which the mass of curcumin was known, was added into a dialysis bag, and the dialysis bag was put in the PBS then. The centrifuge tube was placed in a shaker with 37 °C. At 1, 3, 6, 24 and 72 h, 1 mL PBS containing the released curcumin was taken out to measure the absorption to quantify the amount of curcumin released from Cur@Hb, and 1 mL fresh PBS was added into the centrifuge tube. The drug release curve of curcumin was obtained by calculating the ratio of the cumulative released curcumin at different time points, to the initial amount of curcumin.

### Cell culture

Human hepatocellular carcinoma SMMC7721 cells were maintained in High glucose DMEM (Hyclone, China) supplemented with 10% FBS (BC, Australia) in a humidified atmosphere containing 5% CO_2_ at 37 °C. Cells treated with hypoxia were exposed to a steady flow of low-oxygen gas mixture (1% O_2_, 5% CO_2_, 94% N_2_) in a hypoxia workstation (Invivo2 1000, Ruskinn, UK).

### Cytotoxicity assay

Cells were seeded into 96-well plates. After the cells are attached, 100 μL different concentrations of Cur, Hb and Cur@Hb were added. After 24 h incubation under normoxic conditions, 10 μL CCK8 reagent (DOJINDO, China) was added into each well and incubated for 2 h at 37 °C in the dark. The optical density (OD) value per well was measured at 450 nm with a multi-functional microplate reader (BioTek, USA).

### Flow cytometric analysis of cell cycle and apoptosis

For cell cycle analysis, cells were stained in staining buffer (Bi Yuntian, China) for 30 min at 37 °C, then analyzed on a flow cytometer Beckton Dickinson FACScan (BD Biosciences, San Jose, CA, USA). Quantification of apoptotic cells was also performed by BD FACScan using a Annexin-V-PE/7-AAD Apoptosis Detection Kit (KeyGen BioTECH, China).

### 3D VM formation

Matrigel (Collaborative Biomedical) (Corning, USA) was thawed at 4 °C, then 200 μL Matrigel was rapidly added to each well of a 24-well plate. Tumor cells were seeded into Matrigel-coated wells, then incubated in normoxic or hypoxic (1% O_2_) condition at 37 °C for different time.

### Western blot analysis of protein expression

Western blotting was performed using standard procedures. The following primary antibodies were used: Twist1, vascular endothelial cadherin (VE-cadherin), MMP2 and β-actin (Cell Signaling, USA). Experiments were repeated at least 3 times.

### Colony formation assay

Pretreated cells from the add Cur, Hb, Cur@Hb groups and the negative control groups were seeded into 3.5 cm culture dishes. The cells were exposed to 0, 2, 4, 6, and 8 Gy X-ray (160 kV) at room temperature using a linear accelerator (RadSource, Suwanee, GA, USA) at a dose rate of 1.15 Gy/min. After irradiation, control and irradiated cells were incubated for 12 days. Then colonies were stained with 0.1% crystal violet. Then, the cell survival fraction was figured out, and the mean lethal dose (D0) was calculated by the linear quadratic model.

### Immunofluoresence staining for γH2AX-foci

Slides were stained with primary antibody for γH2AX (Epitomics, CA, USA) and incubated with Alexa-488-conjugated anti-rabbit IgG. Visualization of foci was performed by a FV1200 confocal microscope (Olympus, Japan). Every slide was counted at least 3 times by two blinded observers independently.

### Comet assay

After 24 h incubation under normoxic or hypixic conditions, the cells were irradiated with 4 Gy. Thirty minutes, one hour or four hours later, the cells were harvested. For the comet assay, firstly, the cells were suspended. Then, 50 μL of LMA agarose and 5 μL cell suspension were mixed. A total of 55 μL suspension was quickly spread on the slides with pipette tip. Slides were placed at 4 °C in the dark for 10 min. Slides were immersed in 4 °C Lysis Solution for 30–60 min, and then immersed in freshly prepared Neutral Unwinding Solution for 30 min at 4 °C in the dark. The slides are sent to electrophoresis with the condition of 17 V for 50 min. Drain excess Neutral Electrophoresis Buffer and gently immerse slides in DNA Precipitation Solution for 30 min at room temperature. Immerse slides in 70% ethanol for 30 min at room temperature. Dry samples at 37 °C for 10–15 min. Place 100 µl of diluted SYBR® Green I onto each circle of dried agarose and stain 30 min (room temperature) in the dark. Gently tap slide to remove excess SYBR solution and rinse briefly in water. Allow slides to dry completely at 37 °C. View slides by fluorescence microscopy.

### Macrophages polarization

M2 macrophages were generated by pretreating RAW264.7 cells with 20 ng/mL IL-4 (eBioscience) for 24 h. Then add 250 μg/mL Hb, 250 μg/mL Cur@Hb, 2.5 μg/ mL Cur, 100 ng/mL LPS to M2 macrophages. Samples from cells were incubated with rabbit polyclonal CD206 antibody (dilution 1:100, abcam, catalog number ab64693) and rabbit polyclonal CD86 antibody (dilution 1:100, abcam, catalog number ab3523) to label M2 and M1 macrophages. Isotype controls were employed to establish background fluorescence. Data were acquired on a Beckman Coulter FACSVerse (Beckman Coulter, USA) and analyzed by FlowJo software (Tree Star, San Carlos, CA, USA).

### Tumor-bearing mice model and treatment

6–8 weeks old female SPF grade nude mice (Experimental Animals Center of Shanghai Institute of Life Science, Shanghai, China) were subcutaneously injected with 100 μL of 3 × 10^6^ hepatoma cells in the left or right hind limbs. After the tumor volume reached 100 mm^3^, the mice were randomly divided into 8 groups: PBS group and Hb Group, Cur group, Cur@Hb group, PBS + IR group, Hb + IR group, Cur + IR group, Cur@Hb + IR group, 3–5 animals per group. After tail vein injection of PBS, Hb 10 mg/mL, Cur 1 mg/mL, Cur@Hb 10 mg/mL 200 μL, the human hepatoma xenografts were irradiated by 4 Gy X-ray on day 0, day 2, and day 4. Tumor volumes were measured every other day using the formula V (mm^3^) = ½ab^2^, a is the long diameter of the tumor, b is the short diameter of the tumor, and the unit is mm. The mice used for immunohistochemical studies were sacrificed at the end point of the treatment. Specific primary antibodies, including rabbit polyclonal antibody to human HIF-1α, VEGF, Ki-67 (KeyGen Biotech.) and mouse CD31 (eBioscience, Inc.) were used for immunohistochemical staining. Apoptotic cells in tumor tissues were detected by TUNEL staining using an In Situ Cell Death Detection Kit (KeyGen Biotech.). All animal experimental protocols were approved by the Institutional Animal Care and Use Committee of Soochow University and complied with the code of ethics for animal experimentation.

### Statistical analysis

Data were presented as mean ± SD, unless otherwise stated. All statistical parameters were calculated with GraphPad Prism 6.01 (GraphPad Software Inc.). Student’s t test was used for most data analysis. For comparisons among more than two groups, One-way Analysis of Variance (ANOVA) followed by Bonferroni post-test was performed. *P* < 0.05 was considered to be statistically different.

## Supplementary Information

**Additional file 1.** **Figure S1**. Cur@Hb nanoparticles had good dispersion and dispersibility in aqueous solution, PBS, PBS containing 10% FBS and DMEM containing 10% FBS. **Figure S2**. The standard curve and release kinetics of Cur. **Figure S3**. Quantitatively analyzed the photo-acoustic images of Cur@Hb nanoparticles in vivo. **Figure S4**. A: The uptake of Cur@Hb nanoparticles by SMMC7721 cells; B and C: Fluorescence peaks measured by flow cytometry in normoxic and hypoxic environments. **Figure S5**. A: The cell cycle detected by flow cytometry after normoxic culture for 24 h; B: The cell cycle detected by flow cytometry after hypoxic culture for 24 h. **Figure S6**. The apoptosis rate of the Cur@Hb group was significantly higher than that of the Cur group and the Hb group under both culture conditions, with or without X-ray irradiation. **Figure S7**. A and B: Cur@Hb could significantly inhibit the migration of tumor cells at 24 h and 48 h under normoxia or hypoxia culture; C and D: Cur@Hb inhibited the formation of lumen-like structures under normoxia or hypoxia culture. **Figure S8**. Cur@Hb nanoparticles not only increased the peak value of γH2AX foci produced by X-ray, but also extended the duration of damage. **Figure S9**. A: Under normoxic culture, Cur@Hb nanoparticles increased the production of ROS in SMMC7721 cells; B: Under normoxic culture, Cur@Hb nanoparticles promoted the polarization of M2 macrophages to M1. Data are representative of three independent experiments and expressed as mean ± SD, one-way ANOVA followed by Bonferroni post-test, *p<0.05, **p<0.01, and ***p<0.001. **Figure S10**. Cur@Hb nanoparticles increased the concentration of TNF-α, almost 1.5 times more than that of control group. **Figure S11**. Photoacoustic imaging pictures of Cur@Hb nanoparticles at different time points. **Figure S12**. Body weight of nude mice. **Figure S13**. Cur@Hb nanoparticles were well-tolerated in animal safety studies, without abnormity in organ histology.

## Data Availability

All data generated or analyzed during this study are included in this published article.
