# Supplementary material for: Robust radiosensitization of hemoglobin-curcumin nanoparticles suppresses hypoxic hepatocellular carcinoma
Source: J Nanobiotechnology. 2022 Mar 5;20:115. doi: 10.1186/s12951-022-01316-w (PMC8898525; doi:10.1186/s12951-022-01316-w)
Supplement: Supplementary file 1 — Additional file 1. Figure S1. Cur@Hb nanoparticles had good dispersion and dispersibility in aqueous solution, PBS, PBS containing 10% FBS and DMEM containing 10% FBS. Figure S2. The standard curve and release kinetics of Cur. Figure S3. Quantitatively analyzed the photo-acoustic images of Cur@Hb nanoparticles in vivo. Figure S4. A: The uptake of Cur@Hb nanoparticles by SMMC7721 cells; B and C: Fluorescence peaks measured by flow cytometry in normoxic and hypoxic environments. Figure S5. A: The cell cycle detected by flow cytometry after normoxic culture for 24 h; B: The cell cycle detected by flow cytometry after hypoxic culture for 24 h. Figure S6. The apoptosis rate of the Cur@Hb group was significantly higher than that of the Cur group and the Hb group under both culture conditions, with or without X-ray irradiation. Figure S7. A and B: Cur@Hb could significantly inhibit the migration of tumor cells at 24 h and 48 h under normoxia or hypoxia culture; C and D: Cur@Hb inhibited the formation of lumen-like structures under normoxia or hypoxia culture. Figure S8. Cur@Hb nanoparticles not only increased the peak value of γH2AX foci produced by X-ray, but also extended the duration of damage. Figure S9. A: Under normoxic culture, Cur@Hb nanoparticles increased the production of ROS in SMMC7721 cells; B: Under normoxic culture, Cur@Hb nanoparticles promoted the polarization of M2 macrophages to M1. Data are representative of three independent experiments and expressed as mean ± SD, one-way ANOVA followed by Bonferroni post-test, *p<0.05, **p<0.01, and ***p<0.001. Figure S10. Cur@Hb nanoparticles increased the concentration of TNF-α, almost 1.5 times more than that of control group. Figure S11. Photoacoustic imaging pictures of Cur@Hb nanoparticles at different time points. Figure S12. Body weight of nude mice. Figure S13. Cur@Hb nanoparticles were well-tolerated in animal safety studies, without abnormity in organ histology. [file 12951_2022_1316_MOESM1_ESM.doc]

**Supporting Information**

**Robust Radiosensitization of Hemoglobin-Curcumin Nanoparticles Suppresses Hypoxic Hepatocellular Carcinoma**

Ruoling Gao, Yuan Gu, Ying Yang, Yuping He, Wenpeng Huang, Ting Sun, Zaixiang Tang,

Yong Wang*, Wei Yang*


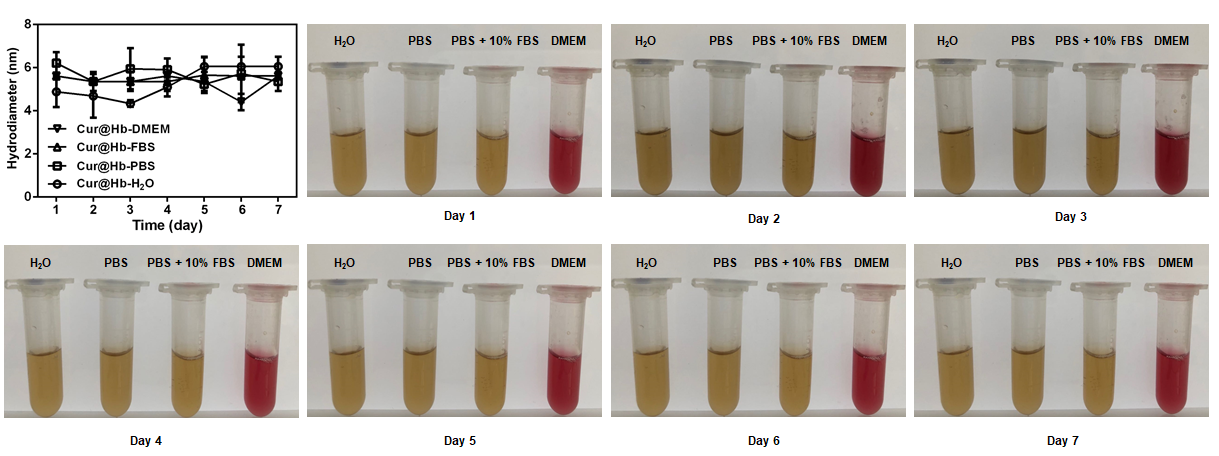


Figure S1. Cur@Hb nanoparticles had good dispersion and dispersibility in aqueous solution, PBS, PBS containing 10% FBS and DMEM containing 10% FBS.


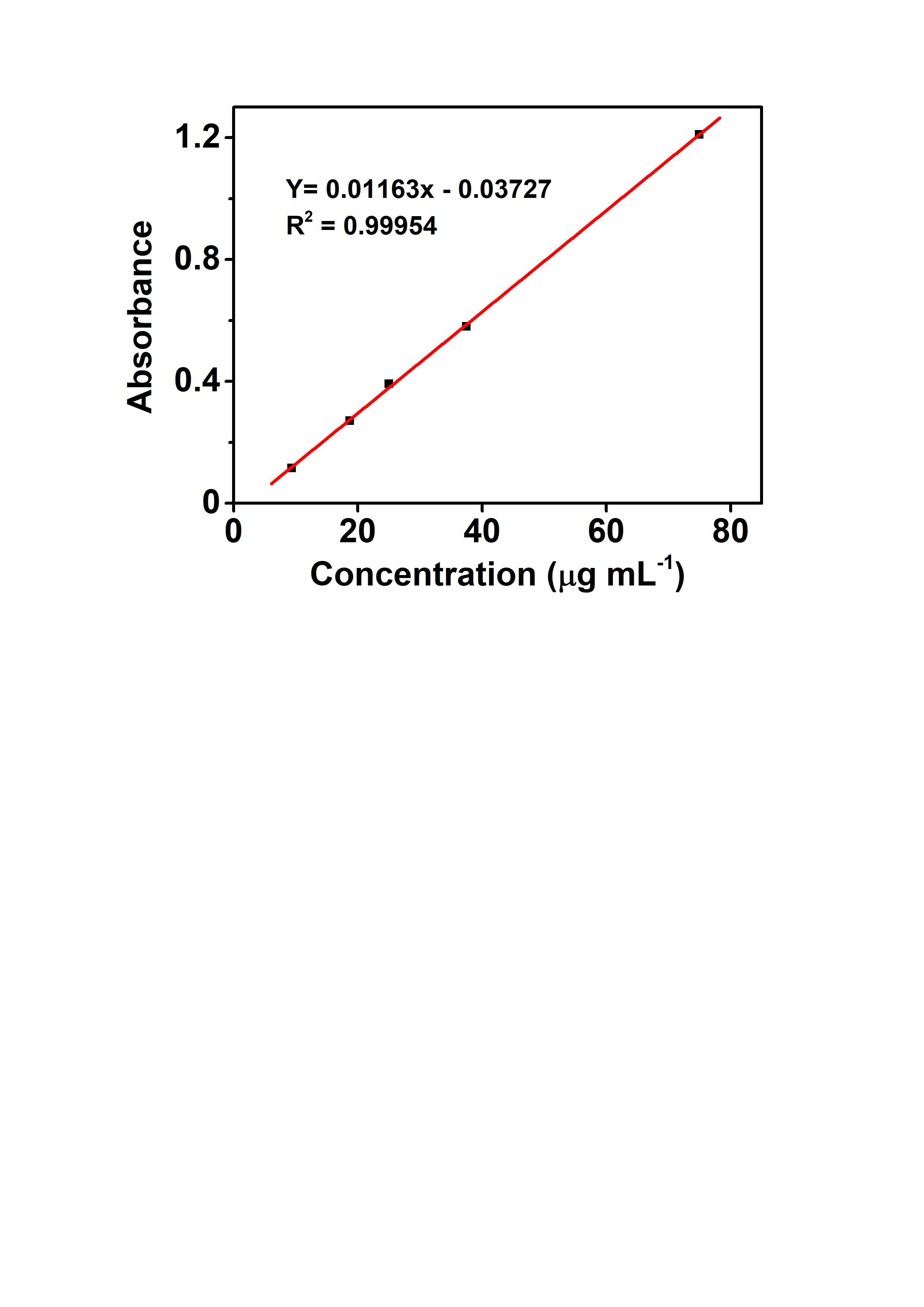

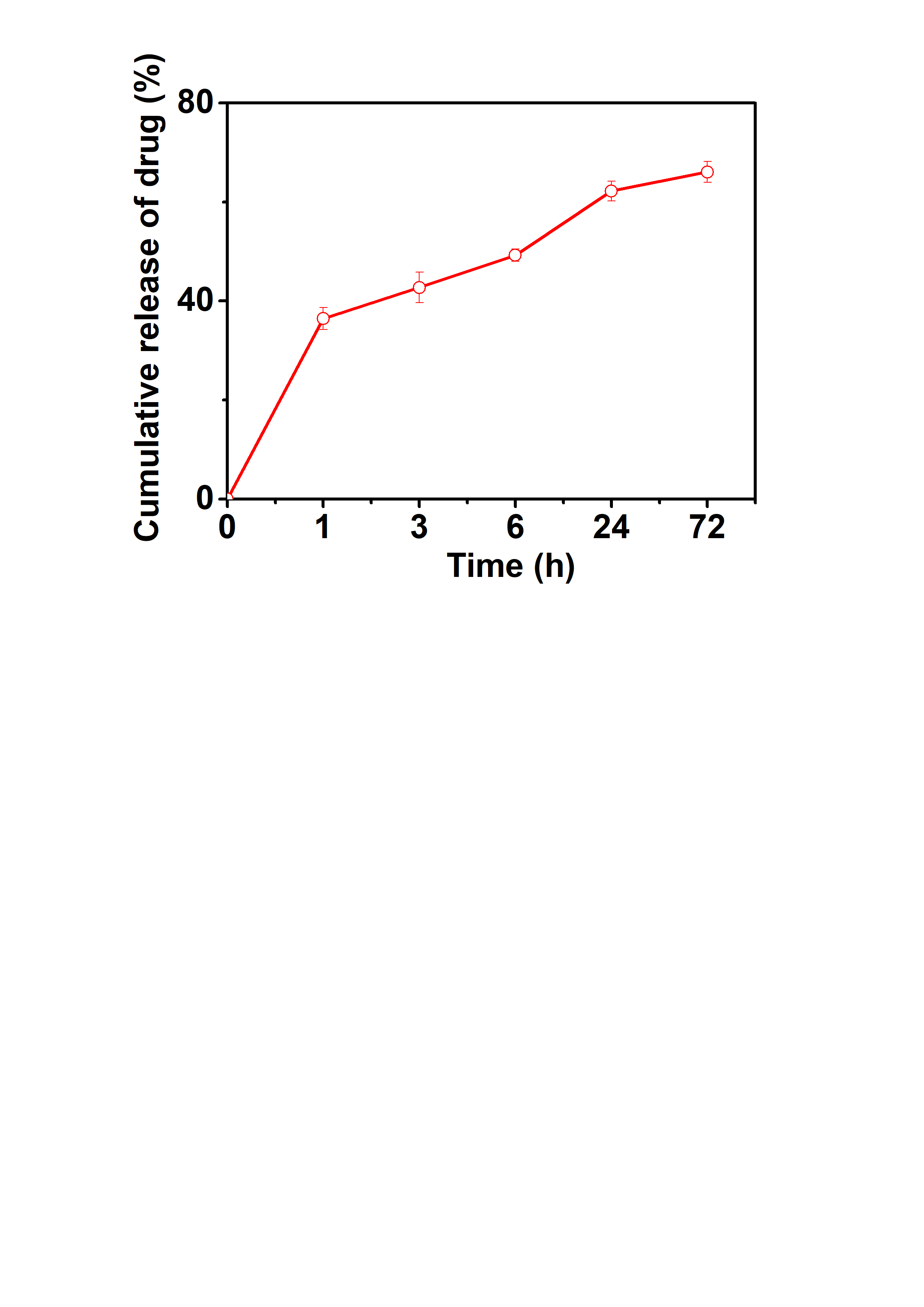


A

B

A. Standard curve of Cur. B. The release kinetics of Cur.

Figure S2. The standard curve and release kinetics of Cur.


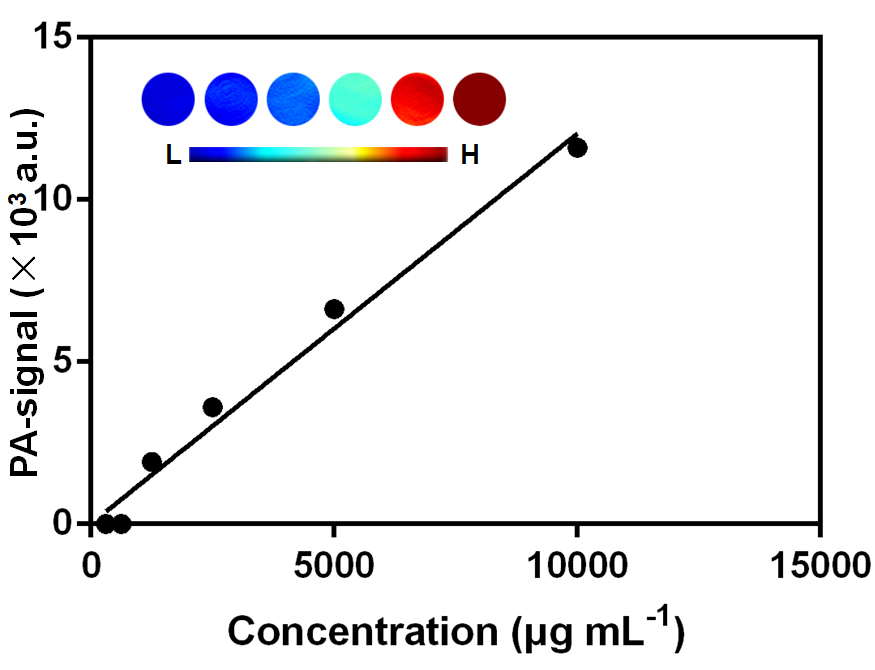


Figure S3. Quantitatively analyzed the photo-acoustic images of Cur@Hb nanoparticles in vivo.

**A**

**Lysosome**

**DAPI**

**Cy-NP**

**Merge**


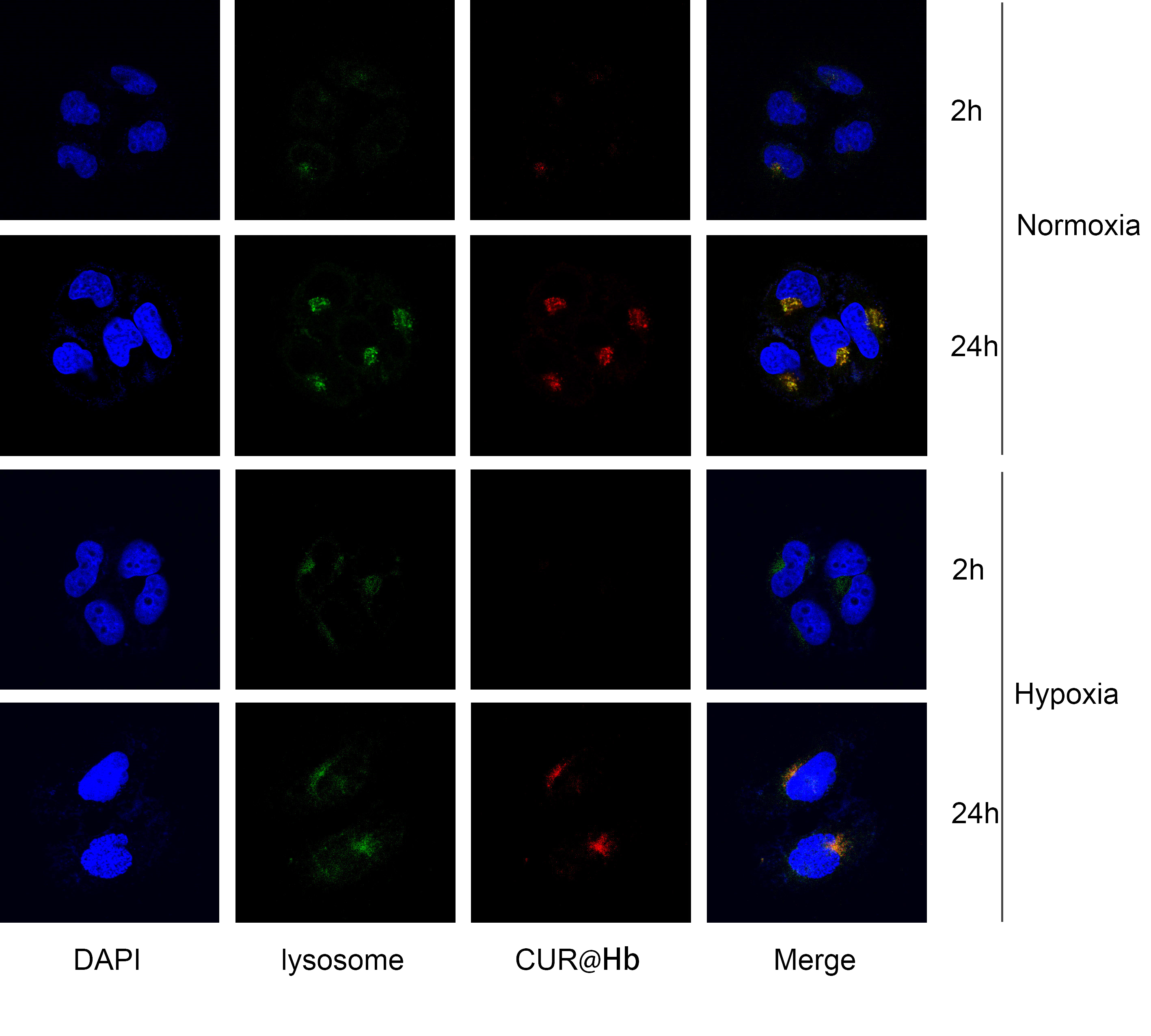


**Normoxia**

**Hypoxia**

**2 h**

**24 h**

**2 h**

**24 h**


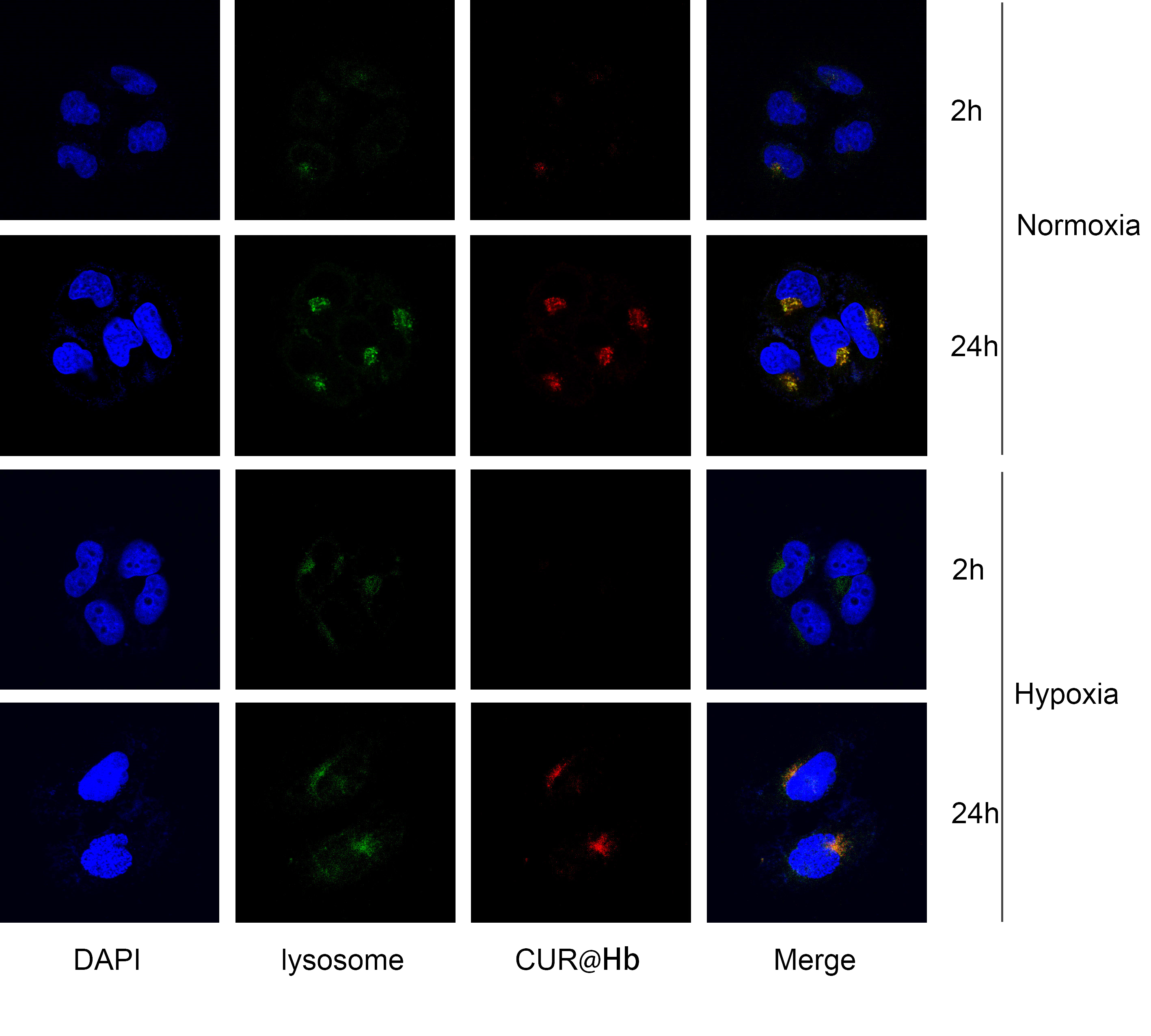

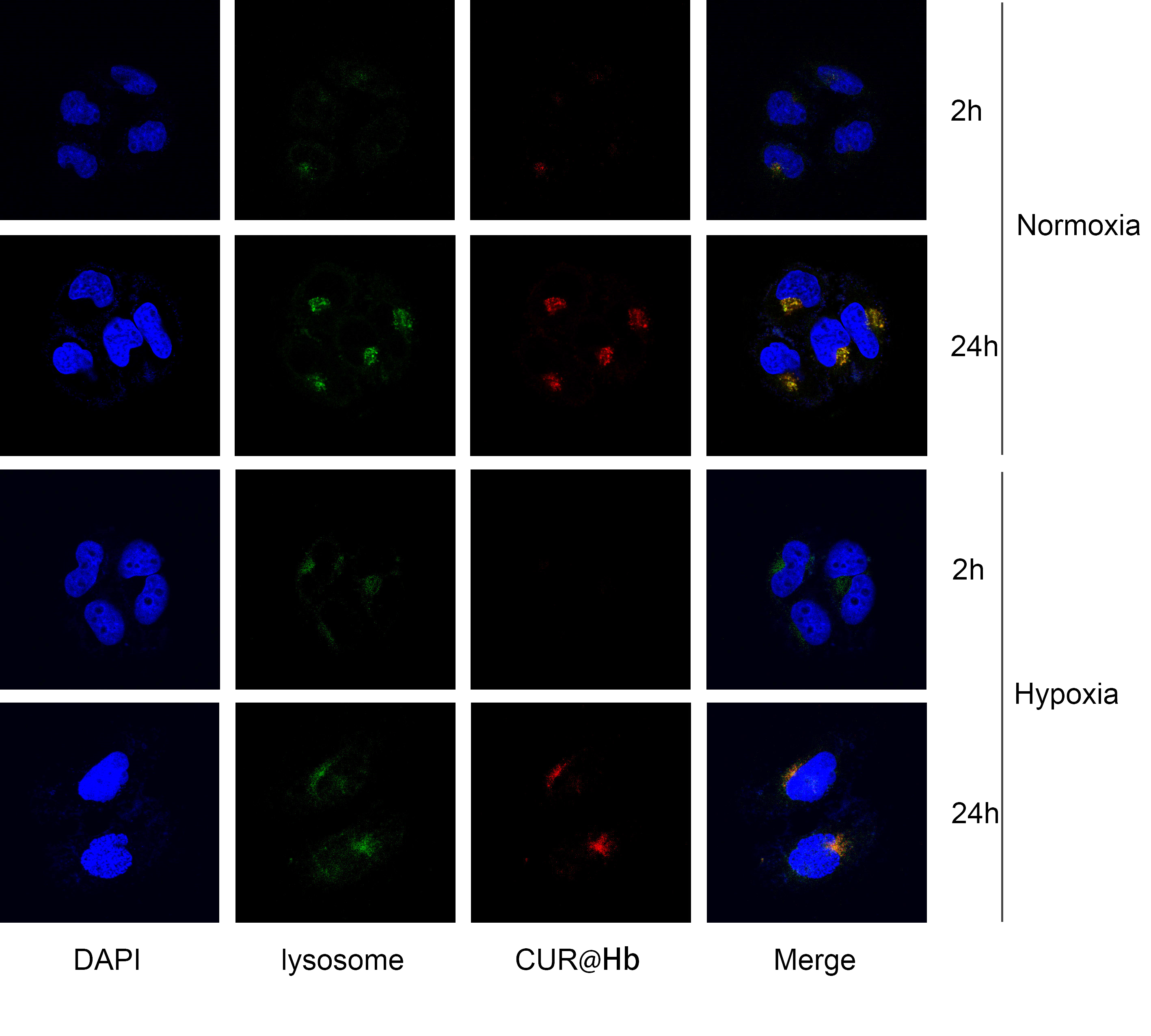

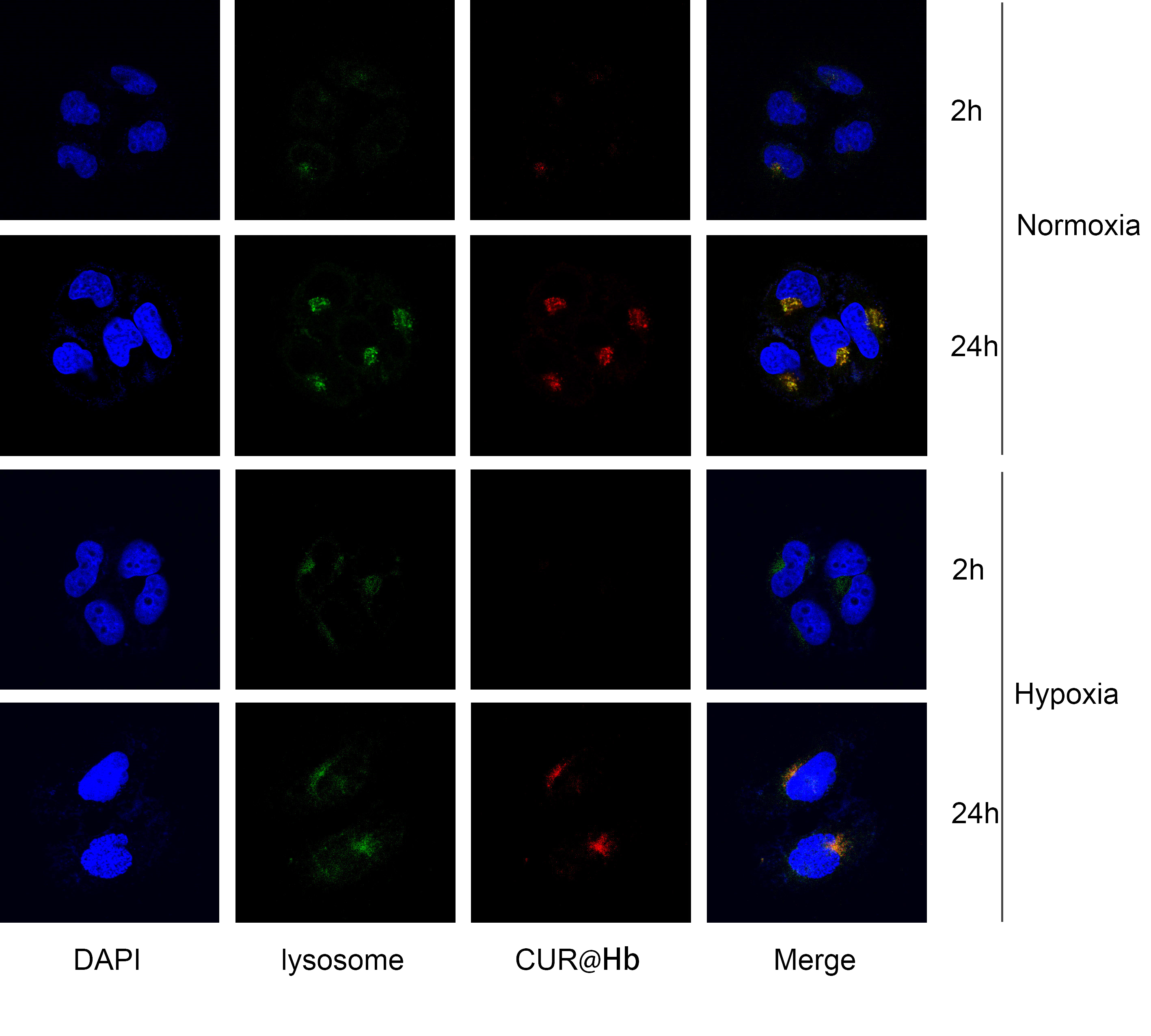


**B C**

**
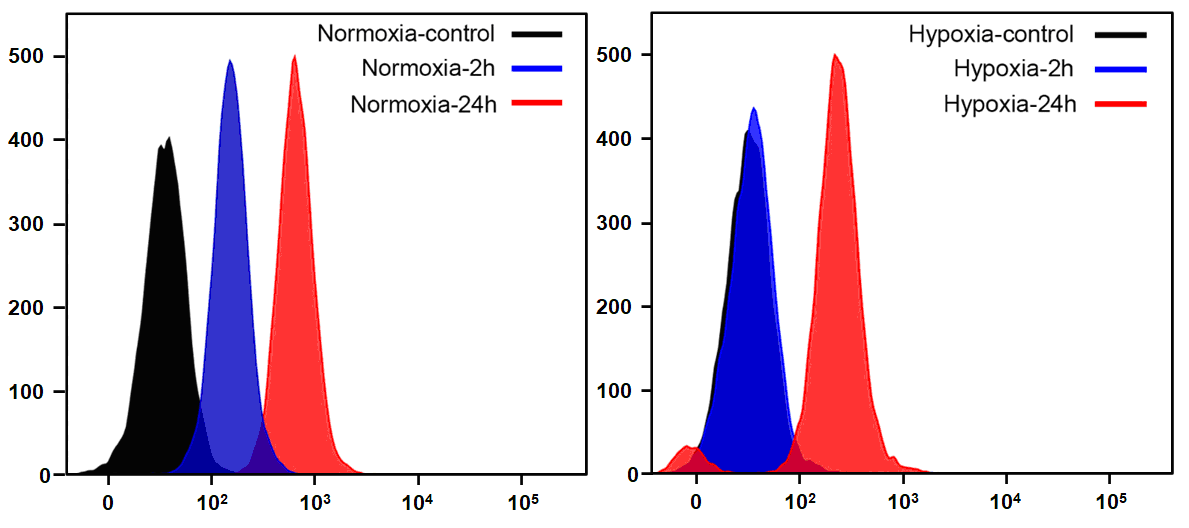
**

Figure S4. A: The uptake of Cur@Hb nanoparticles by SMMC7721 cells; B and C: Fluorescence peaks measured by flow cytometry in normoxic and hypoxic environments.


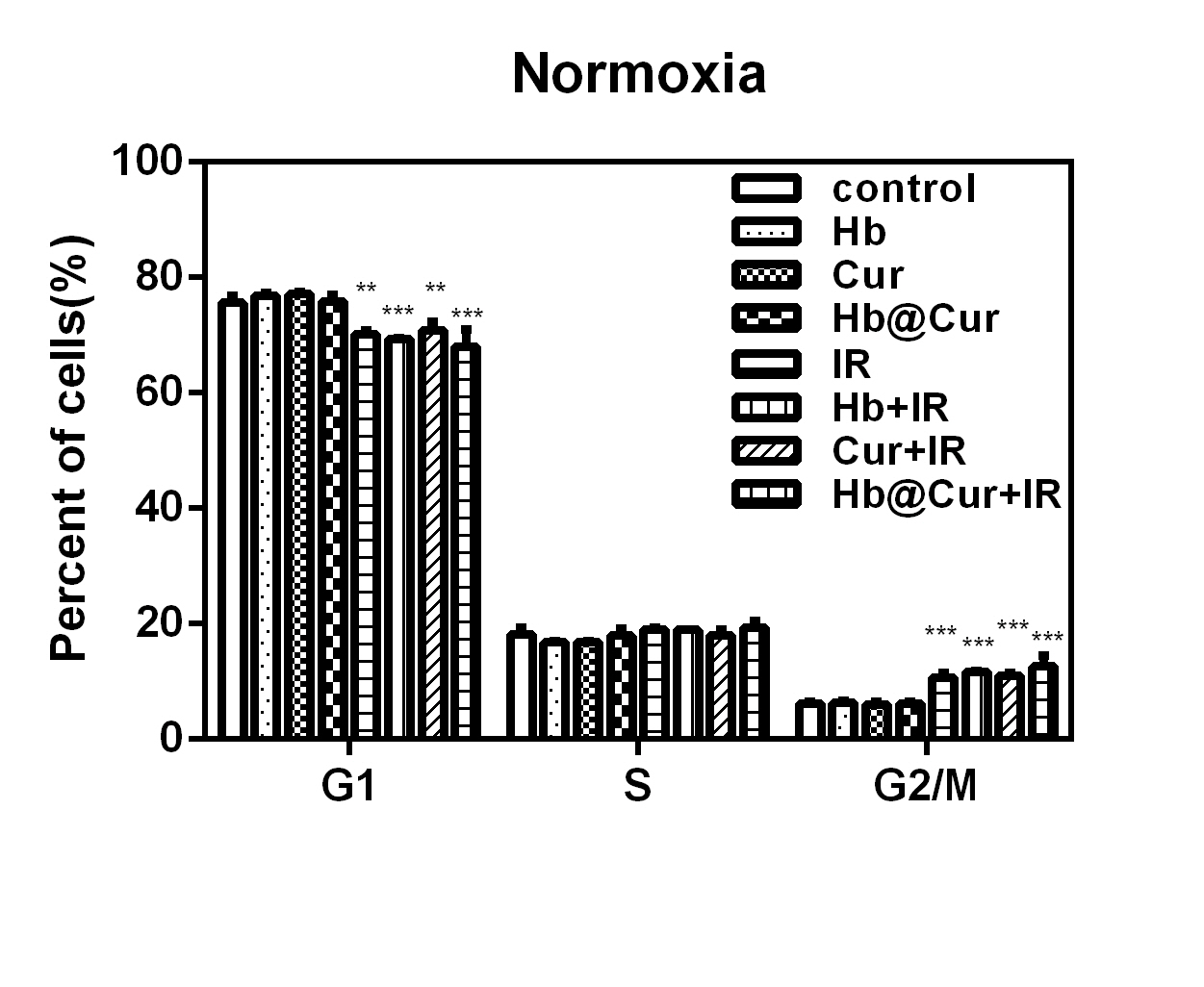


**Normoxia**

**Percent of cells (%)**

**Normoxia**

**Hypoxia**

**Percent of cells (%)**


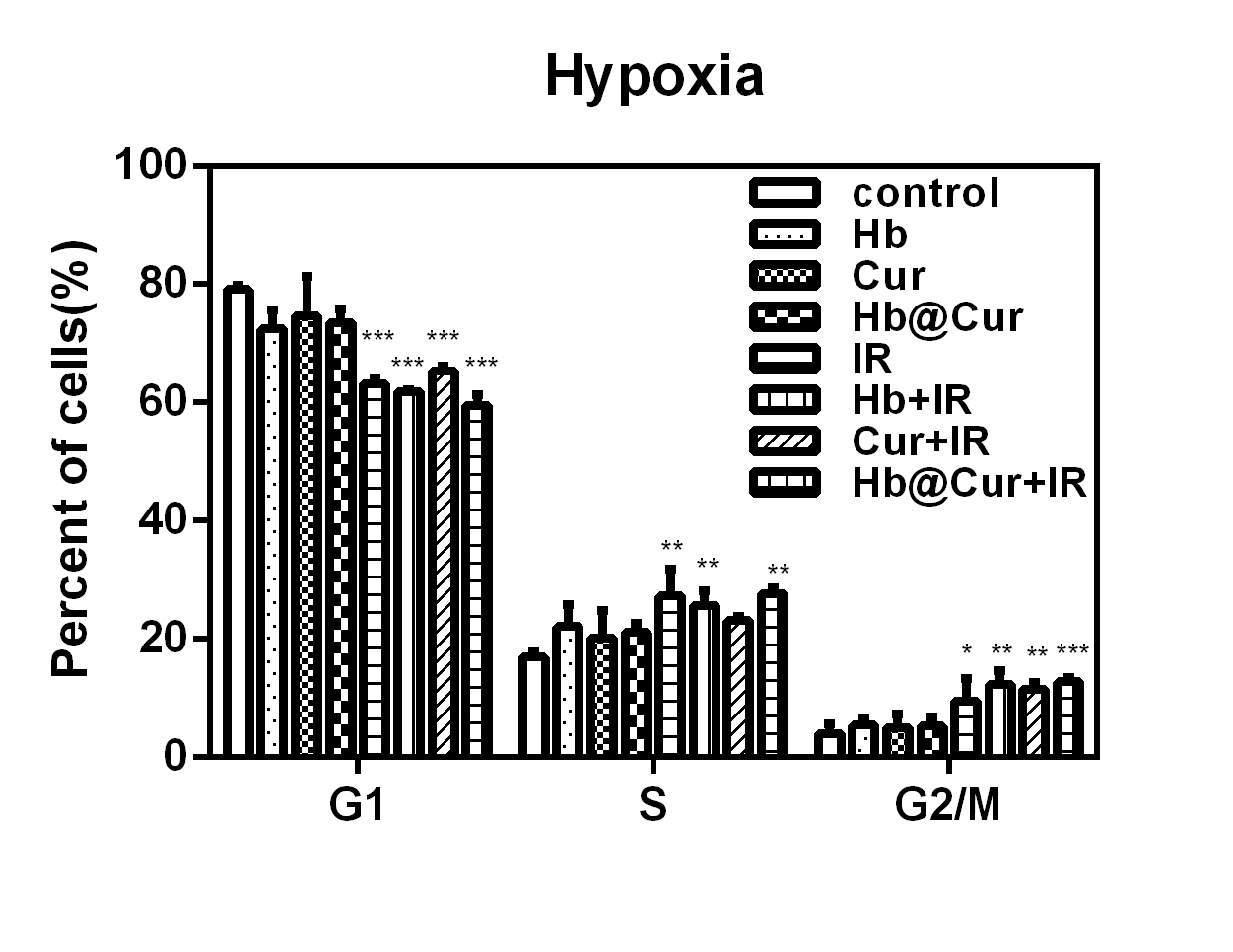


Figure S5. A: The cell cycle detected by flow cytometry after normoxic culture for 24 h; B: The cell cycle detected by flow cytometry after hypoxic culture for 24 h.


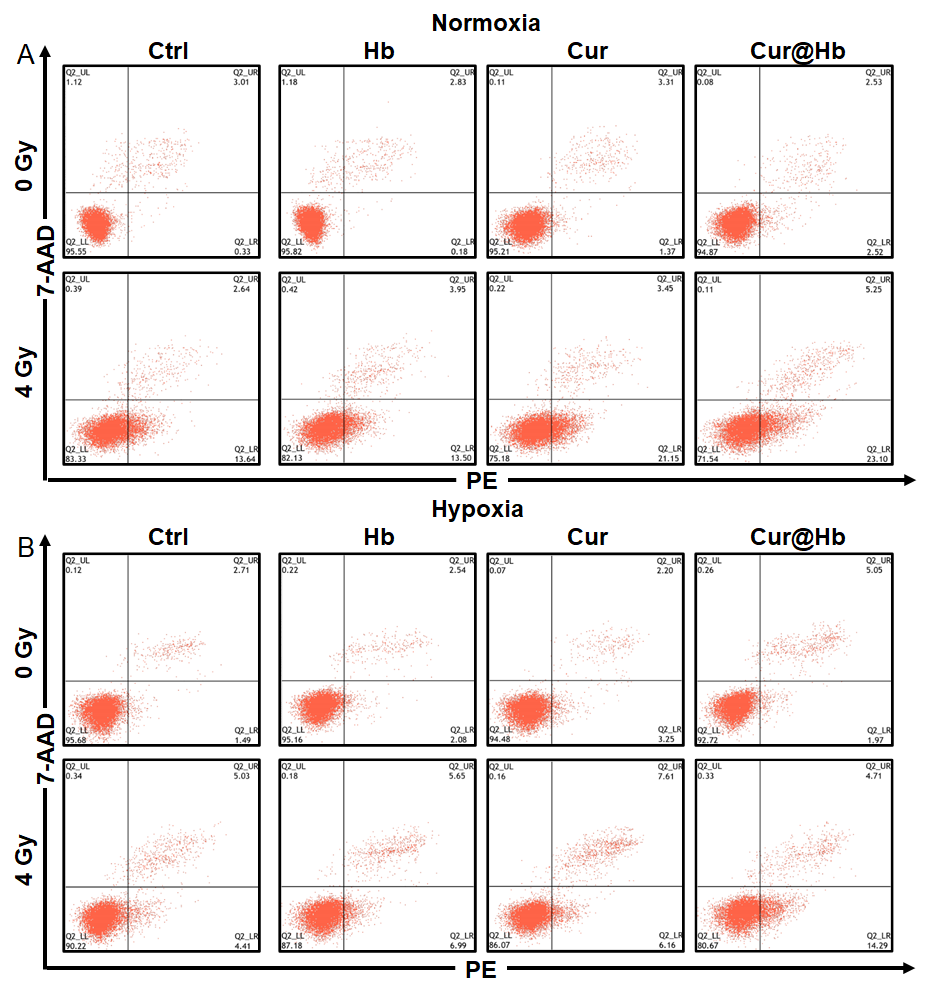


Figure S6. The apoptosis rate of the Cur@Hb group was significantly higher than that of the Cur group and the Hb group under both culture conditions, with or without X-ray irradiation.

**A**

**Normoxia**


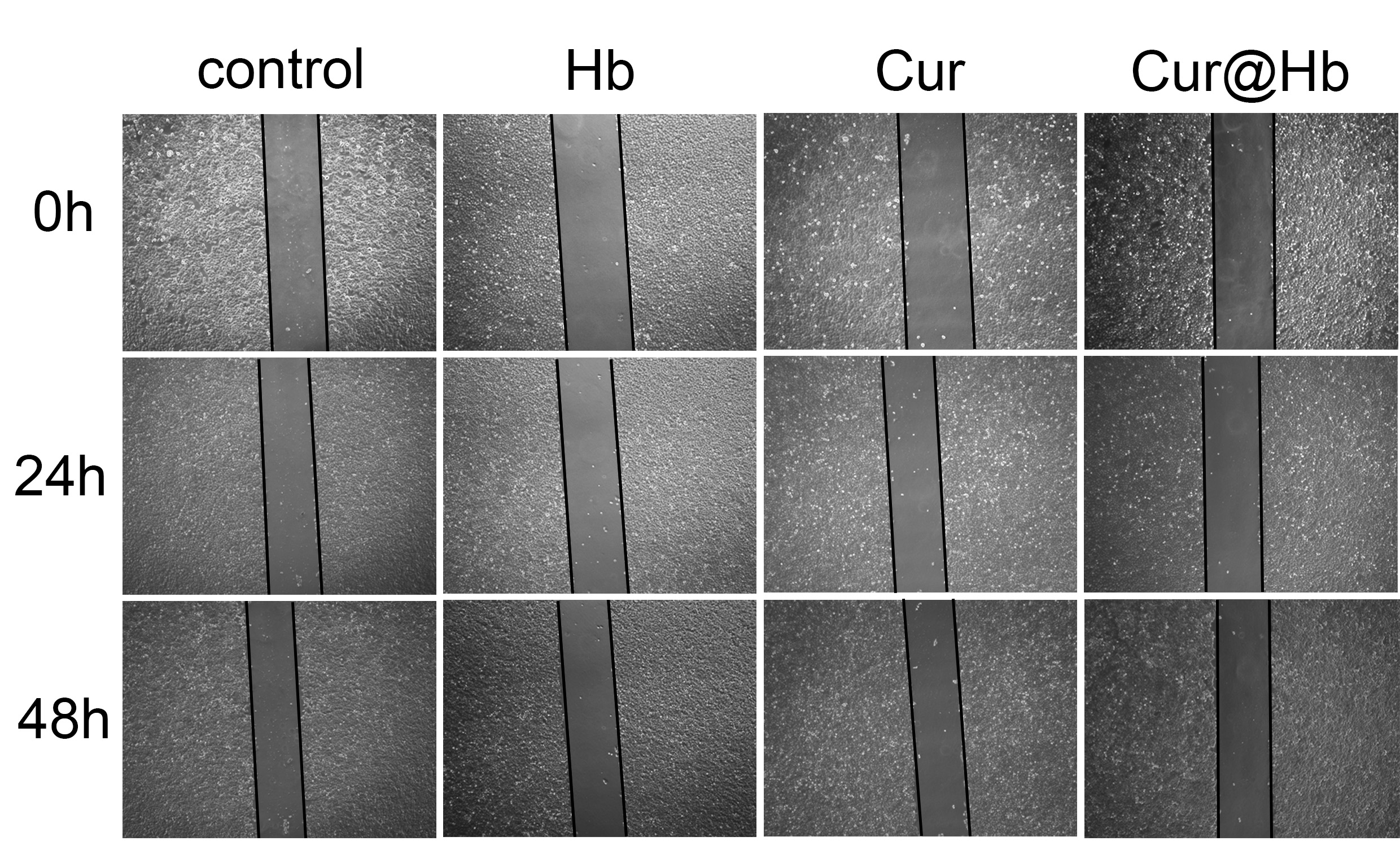


**0 h**

**24 h**

**48 h**

**Ctrl**

**Hb**

**Cur**

**Cur@Hb**

**B**

**Hypoxia**


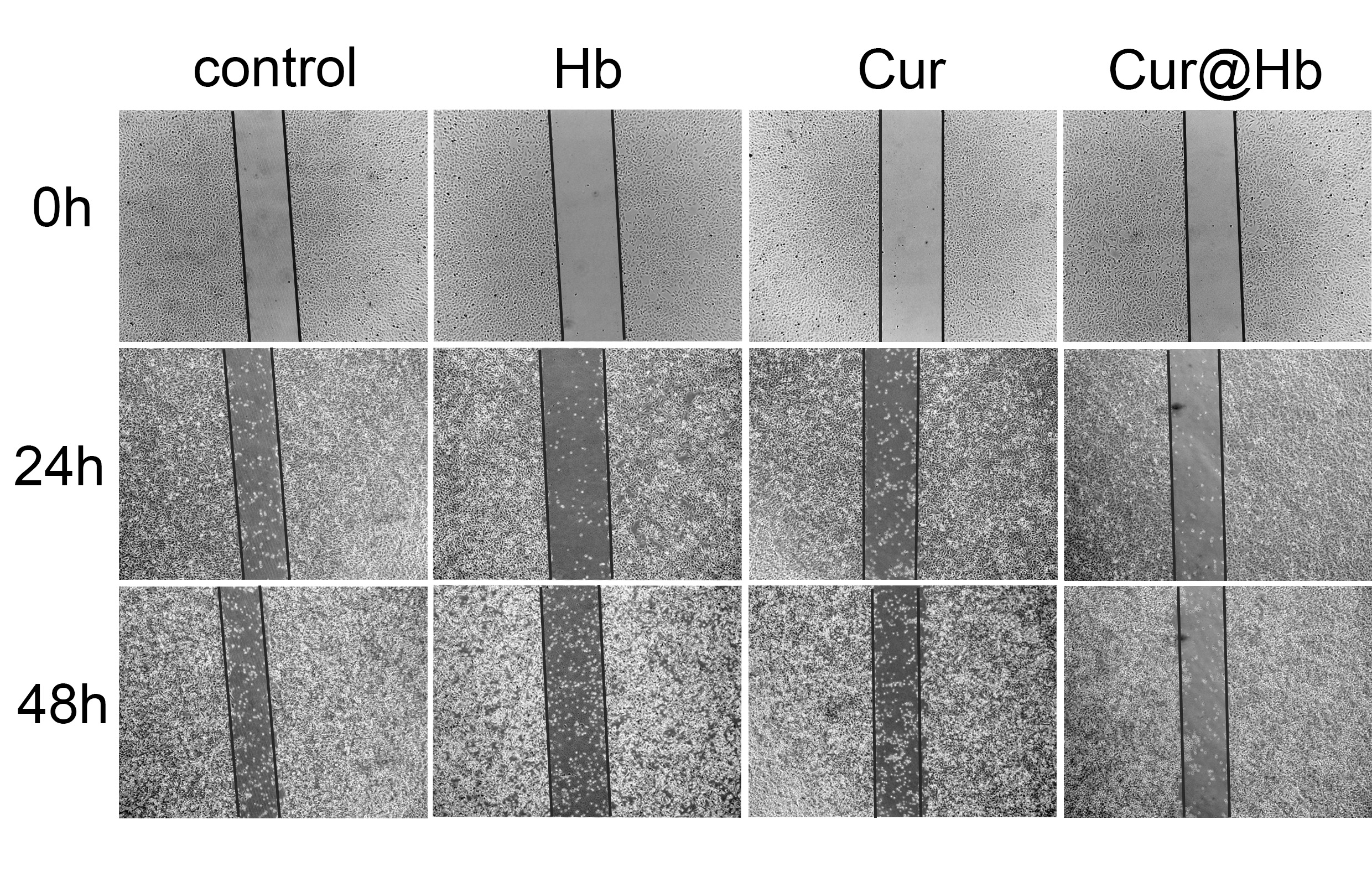


**0 h**

**24 h**

**48 h**

**Ctrl**

**Hb**

**Cur**

**Cur@Hb**

**C**

**Normoxia**


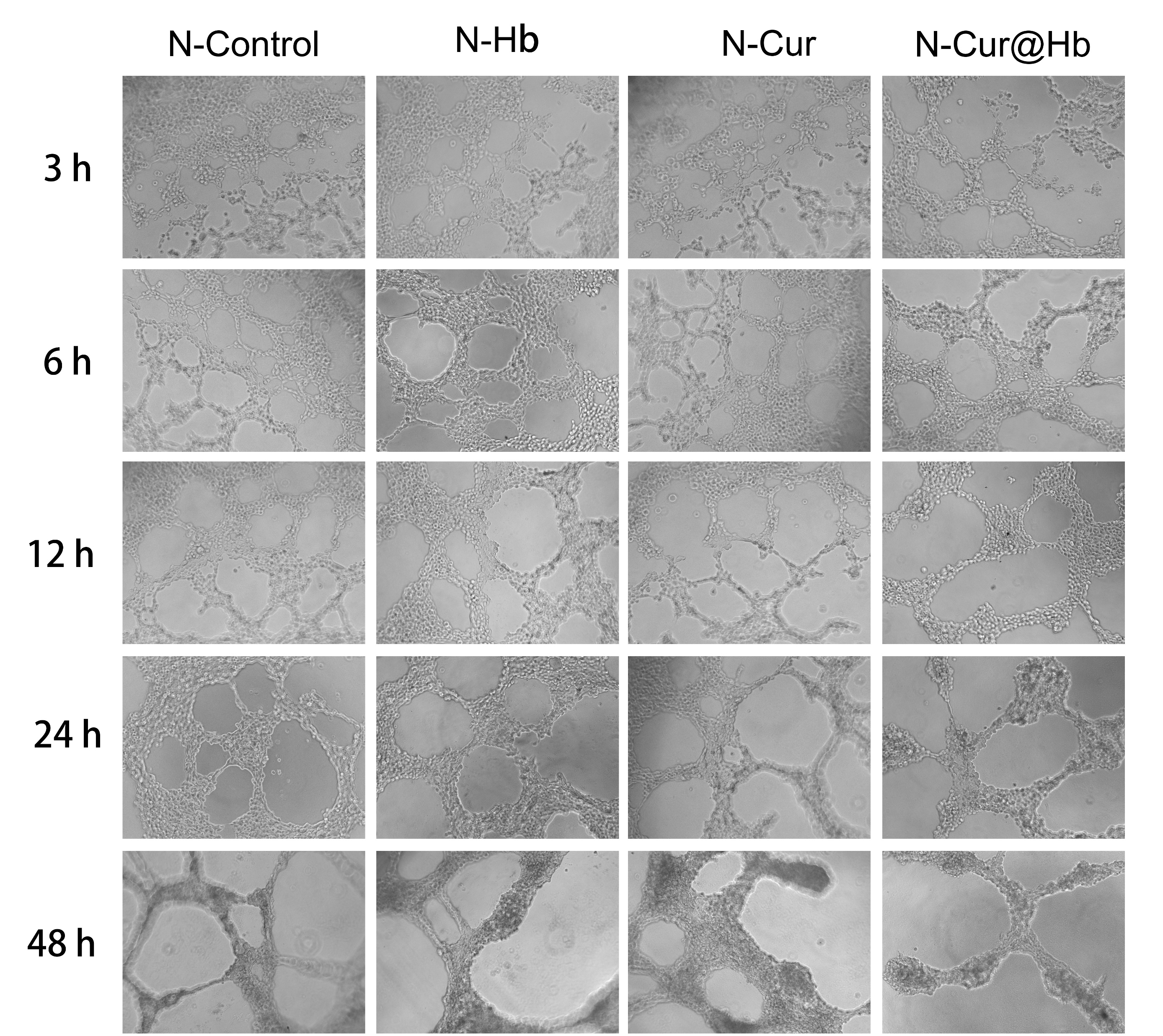


**Ctrl**

**Hb**

**Cur**

**Cur@Hb**

**3 h**

**6 h**

**12 h**

**24 h**

**48 h**

**D**

**Hypoxia**


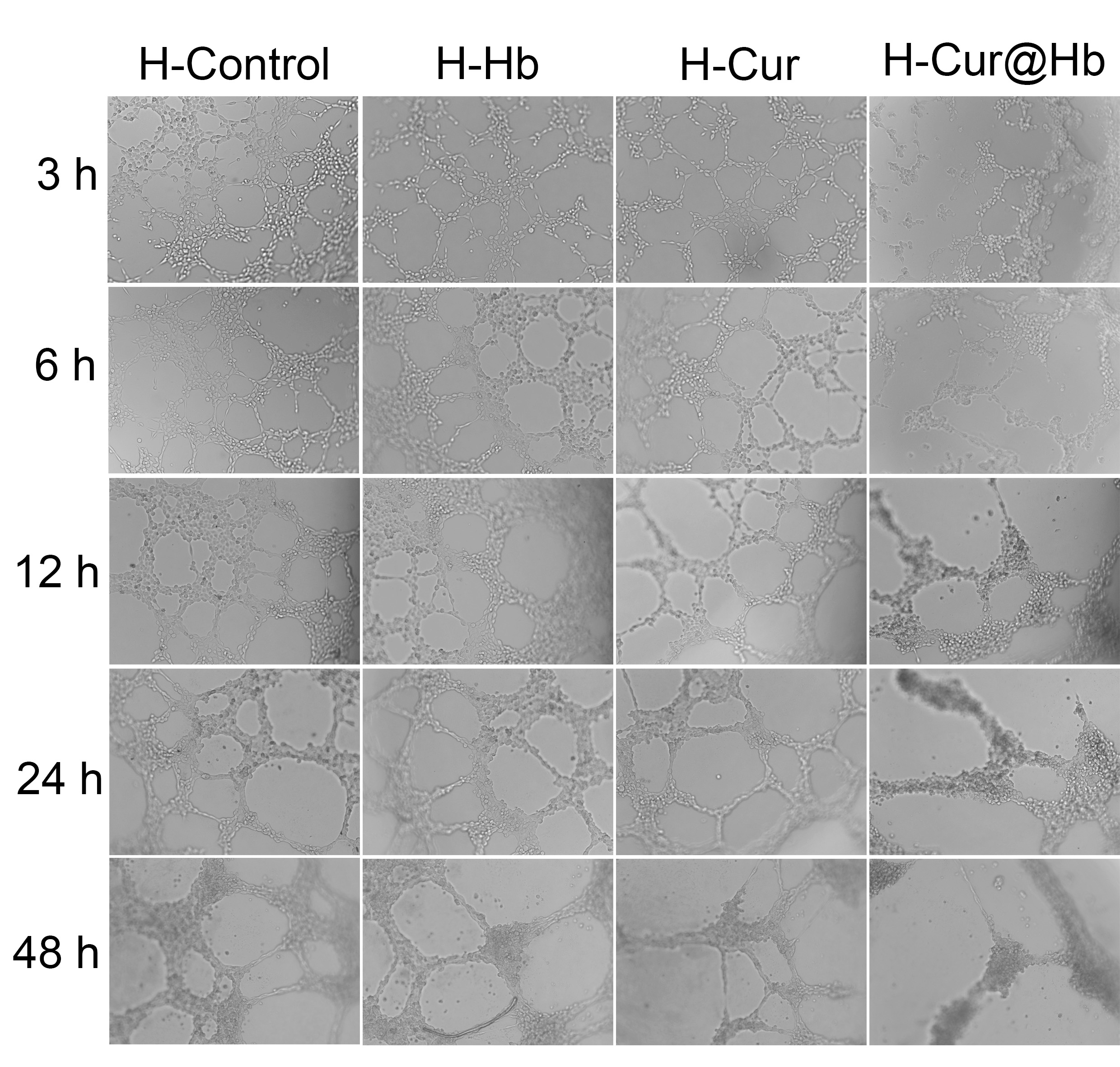


**Ctrl**

**Hb**

**Cur**

**Cur@Hb**

**3 h**

**6 h**

**12 h**

**24 h**

**48 h**

Figure S7. A and B: Cur@Hb could significantly inhibit the migration of tumor cells at 24 h and 48 h under normoxia or hypoxia culture; C and D: Cur@Hb inhibited the formation of lumen-like structures under normoxia or hypoxia culture.

**Normoxia**


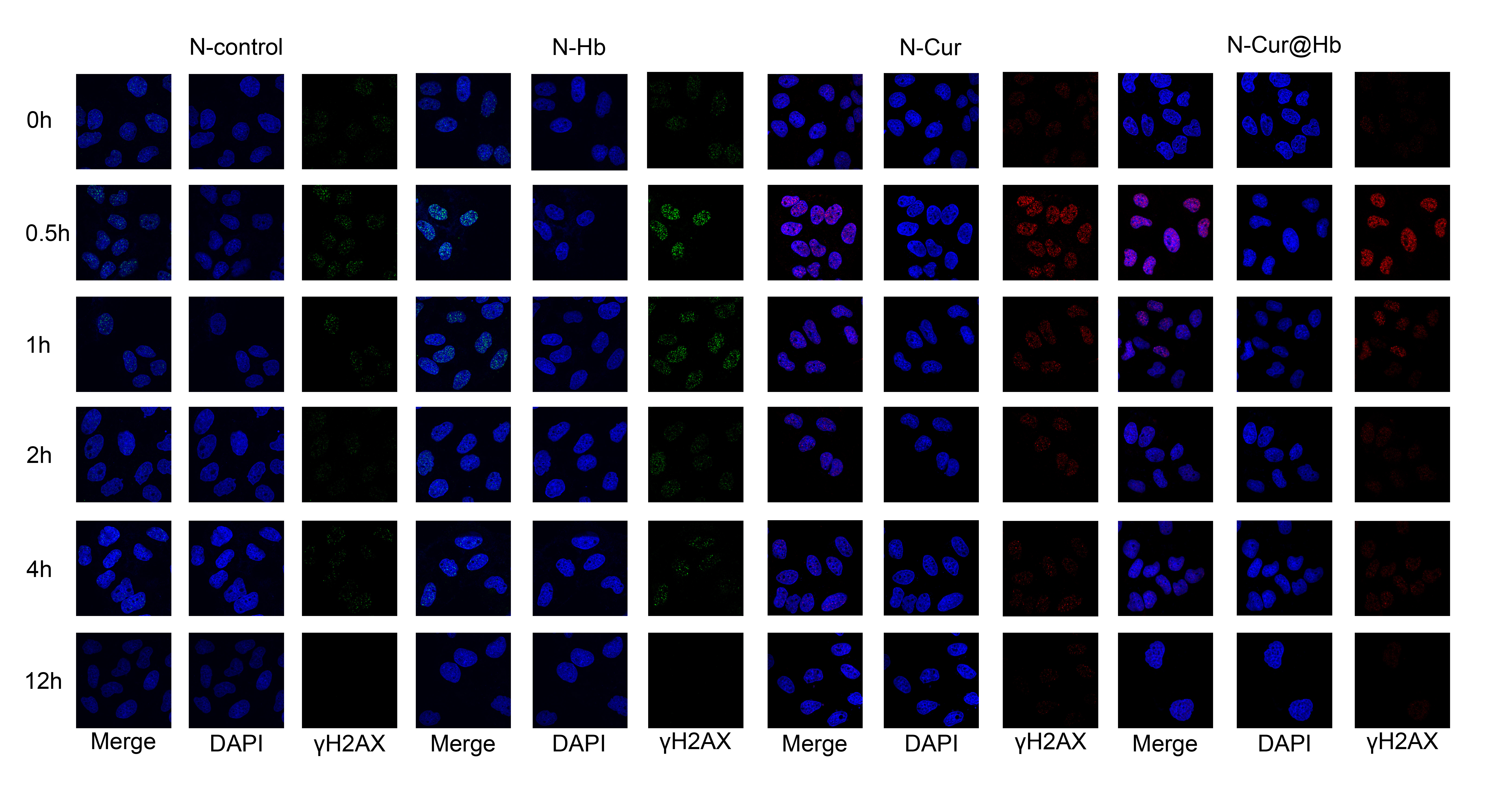


**Ctrl**

**Hb**

**Cur**

**Cur@Hb**

**Merge**

**DAPI**

**γH2AX**

**Merge**

**DAPI**

**γH2AX**

**Merge**

**DAPI**

**γH2AX**

**Merge**

**DAPI**

**γH2AX**

**0.5**

**1**

**4**

**0**

**2**

**12**

A


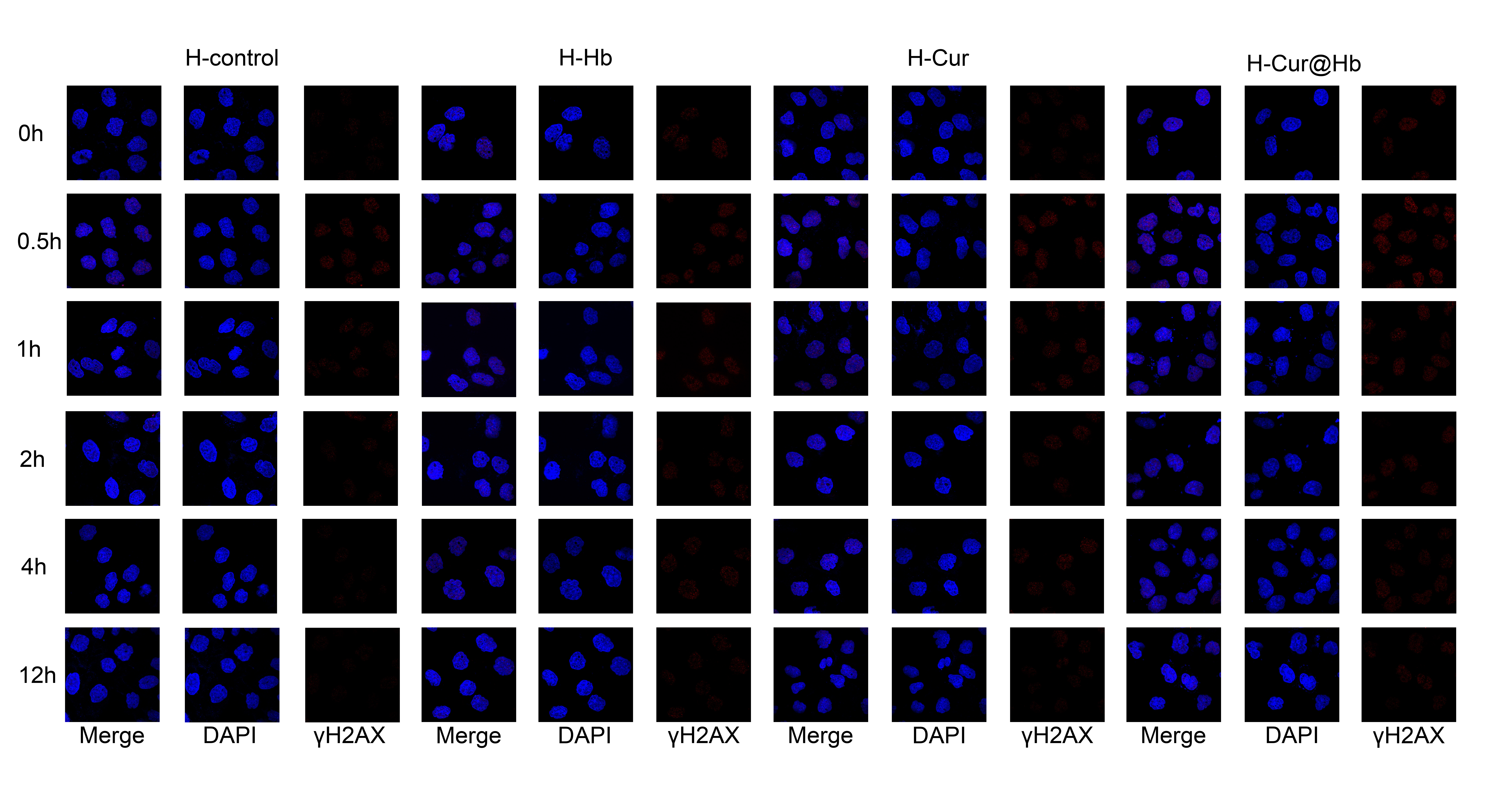


**Hypoxia**

**Ctrl**

**Hb**

**Cur**

**Cur@Hb**

**0.5**

**1**

**4**

**0**

**2**

**12**

**Merge**

**DAPI**

**γH2AX**

**Merge**

**DAPI**

**γH2AX**

**Merge**

**DAPI**

**γH2AX**

**Merge**

**DAPI**

**γH2AX**

Figure S8. Cur@Hb nanoparticles not only increased the peak value of γH2AX foci produced by X-ray, but also extended the duration of damage.


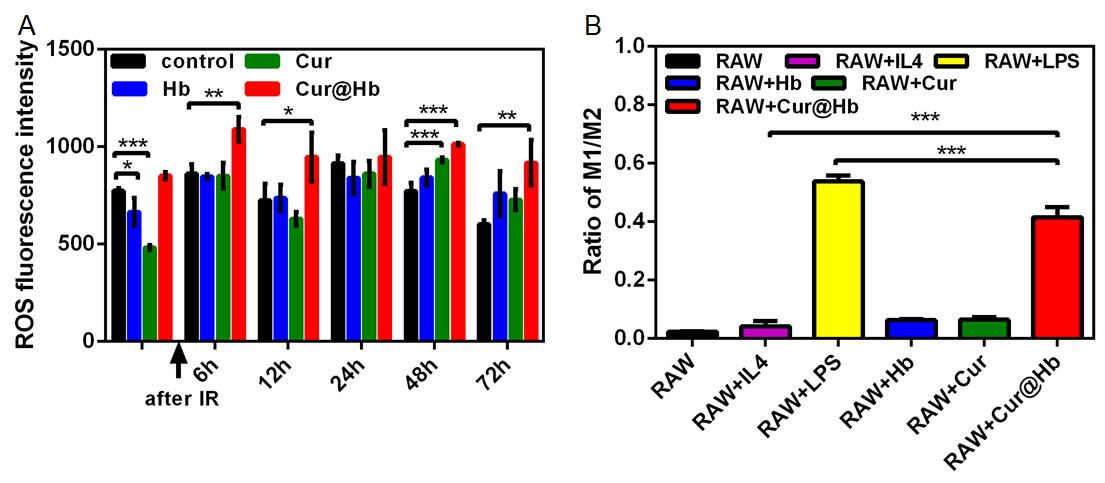


Figure S9. A: Under normoxic culture, Cur@Hb nanoparticles increased the production of ROS in SMMC7721 cells; B: Under normoxic culture, Cur@Hb nanoparticles promoted the polarization of M2 macrophages to M1. Data are representative of three independent experiments and expressed as mean ± SD, one-way ANOVA followed by Bonferroni post-test, **p*<0.05, ***p*<0.01, and ****p*<0.001.


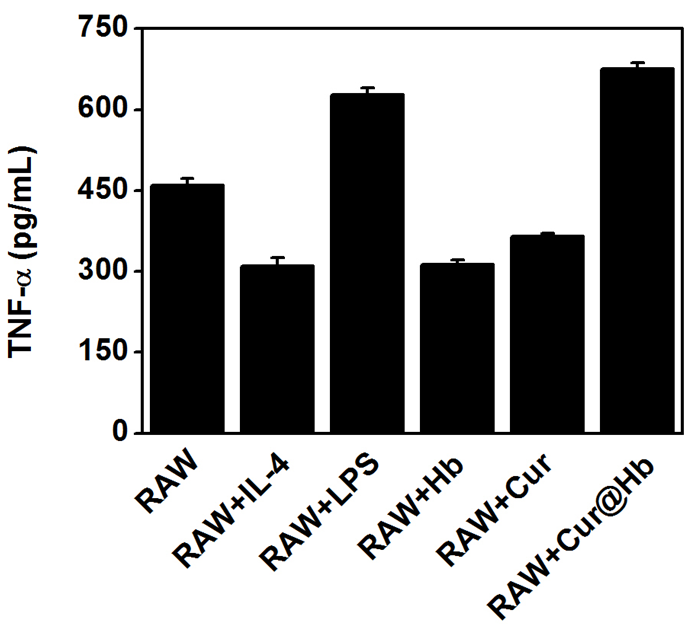


Figure S10. Cur@Hb nanoparticles increased the concentration of TNF-α, almost 1.5 times more than that of control group.


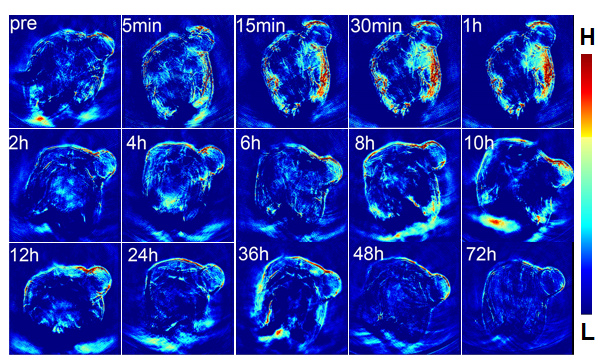


Figure S11. Photoacoustic imaging pictures of Cur@Hb nanoparticles at different time points.


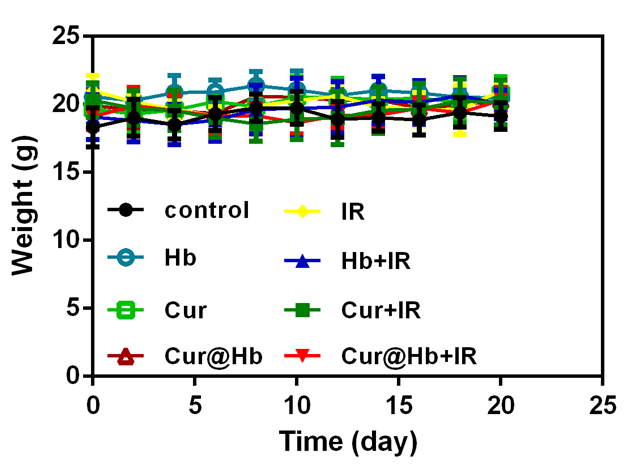


Figure S12. Body weight of nude mice.


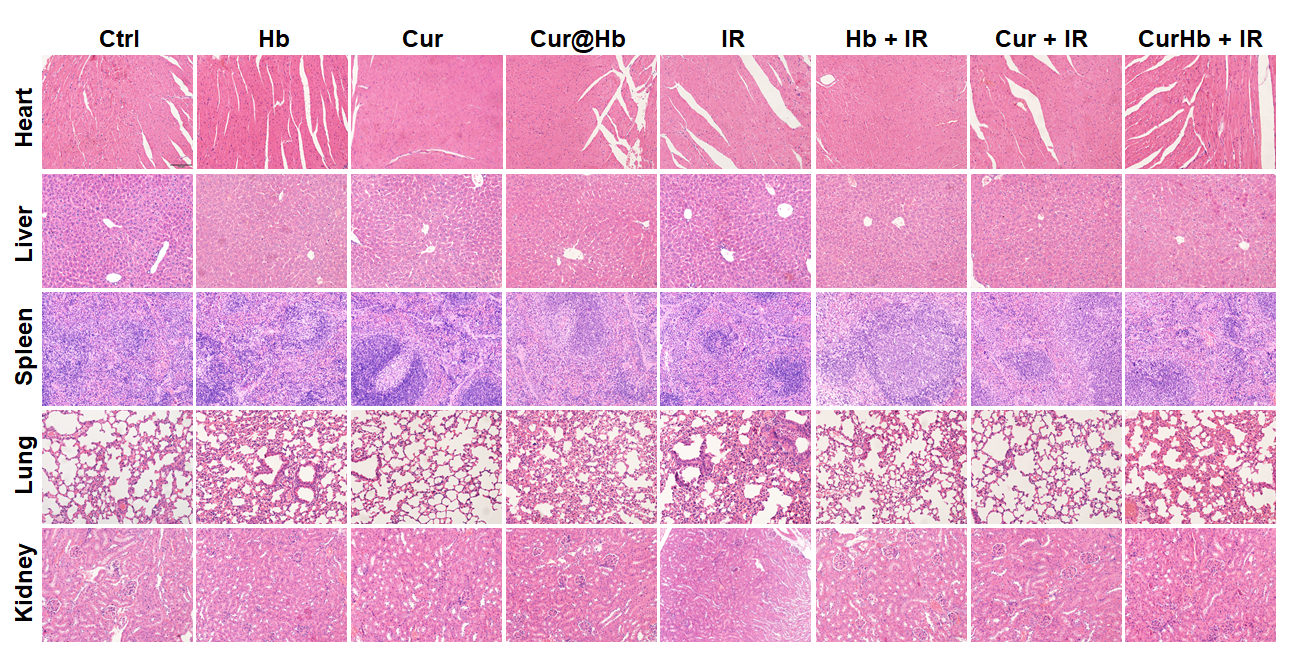


Figure S13. Cur@Hb nanoparticles were well-tolerated in animal safety studies, without abnormity in organ histology.
